# Empiric Methods to Account for Pre-analytical Variability in Digital Histopathology in Frontotemporal Lobar Degeneration

**DOI:** 10.3389/fnins.2019.00682

**Published:** 2019-07-03

**Authors:** Lucia A. A. Giannini, Sharon X. Xie, Claire Peterson, Cecilia Zhou, Edward B. Lee, David A. Wolk, Murray Grossman, John Q. Trojanowski, Corey T. McMillan, David J. Irwin

**Affiliations:** ^1^Penn Digital Neuropathology Laboratory, Department of Neurology, Perelman School of Medicine, University of Pennsylvania, Philadelphia, PA, United States; ^2^Penn Frontotemporal Degeneration Center, Department of Neurology, Perelman School of Medicine, University of Pennsylvania, Philadelphia, PA, United States; ^3^Department of Neurology, University Medical Center Groningen – University of Groningen, Groningen, Netherlands; ^4^Department of Biostatistics, Epidemiology and Informatics, Perelman School of Medicine, University of Pennsylvania, Philadelphia, PA, United States; ^5^Translational Neuropathology Research Laboratory, Department of Pathology and Laboratory Medicine, Perelman School of Medicine, University of Pennsylvania, Philadelphia, PA, United States; ^6^Alzheimer’s Disease Center, Department of Neurology, Perelman School of Medicine, University of Pennsylvania, Philadelphia, PA, United States; ^7^Center for Neurodegenerative Disease Research, Department of Pathology and Laboratory Medicine, Perelman School of Medicine, University of Pennsylvania, Philadelphia, PA, United States

**Keywords:** digital histopathology, frontotemporal lobar degeneration, pre-analytical variability, batch effects, linear transformation method, validation of a method

## Abstract

Digital pathology is increasingly prominent in neurodegenerative disease research, but variability in immunohistochemical staining intensity between staining batches prevents large-scale comparative studies. Here we provide a statistically rigorous method to account for staining batch effects in a large sample of brain tissue with frontotemporal lobar degeneration with tau inclusions (FTLD-Tau, *N* = 39) or TDP-43 inclusions (FTLD-TDP, *N* = 53). We analyzed the relationship between duplicate measurements of digital pathology, i.e., percent area occupied by pathology (%AO) for grey matter (GM) and white matter (WM), from two distinct staining batches. We found a significant difference in duplicate measurements from distinct staining batches in FTLD-Tau (mean difference: GM = 1.13 ± 0.44, WM = 1.28 ± 0.56; *p* < 0.001) and FTLD-TDP (GM = 0.95 ± 0.66, WM = 0.90 ± 0.77; *p* < 0.001), and these measurements were linearly related (R-squared [Rsq]: FTLD-Tau GM = 0.92, WM = 0.92; FTLD-TDP GM = 0.75, WM = 0.78; *p* < 0.001 all). We therefore used linear regression to transform %AO from distinct staining batches into equivalent values. Using a train-test set design, we examined transformation prerequisites (i.e., Rsq) from linear-modeling in training sets, and we applied equivalence factors (i.e., beta, intercept) to independent testing sets to determine transformation outcomes (i.e., intraclass correlation coefficient [ICC]). First, random iterations (×100) of linear regression showed that smaller training sets (*N* = 12–24), feasible for prospective use, have acceptable transformation prerequisites (mean Rsq: FTLD-Tau ≥0.9; FTLD-TDP ≥0.7). When cross-validated on independent complementary testing sets, in FTLD-Tau, *N* = 12 training sets resulted in 100% of GM and WM transformations with optimal transformation outcomes (ICC ≥ 0.8), while in FTLD-TDP *N* = 24 training sets resulted in optimal ICC in testing sets (GM = 72%, WM = 98%). We therefore propose training sets of *N* = 12 in FTLD-Tau and *N* = 24 in FTLD-TDP for prospective transformations. Finally, the transformation enabled us to significantly reduce batch-related difference in duplicate measurements in FTLD-Tau (GM/WM: *p* < 0.001 both) and FTLD-TDP (GM/WM: *p* < 0.001 both), and to decrease the necessary sample size estimated in a power analysis in FTLD-Tau (GM:-40%; WM: -34%) and FTLD-TDP (GM: -20%; WM: -30%). Finally, we tested generalizability of our approach using a second, open-source, image analysis platform and found similar results. We concluded that a small sample of tissue stained in duplicate can be used to account for pre-analytical variability such as staining batch effects, thereby improving methods for future studies.

## Introduction

Digital pathology is emerging as an important discipline in clinical pathology, biomedical research and medical education (Huisman, 2012; Hamilton et al., 2014; Griffin and Treanor, 2017). Digital methods of pathological analysis are also increasingly used in neurodegenerative disease research as they provide a high-throughput, objective measure of disease severity as compared with traditional ordinal ratings. Indeed, this quantitative approach to measuring pathological burden provides increased sensitivity to detect clinicopathological correlations (Neltner et al., 2012; Walker et al., 2015, 2017; Irwin et al., 2016b; Ferman et al., 2018), which are critical to improve the *antemortem* diagnosis of neurodegenerative diseases. This is especially important in less common, heterogeneous disorders such as frontotemporal lobar degeneration (FTLD) (Irwin et al., 2015).

Frontotemporal lobar degeneration comprises a heterogeneous group of neuropathologies, which present clinically as frontotemporal dementia syndromes (Irwin et al., 2015). The two most common FTLD neuropathologies are frontotemporal lobar degeneration with inclusions of the protein tau (FTLD-Tau) and frontotemporal lobar degeneration with inclusions of the transactive response DNA-binding protein of 43 kDa (FTLD-TDP) (Mackenzie et al., 2010; Irwin et al., 2015). FTLD-Tau can be classified into different neuropathological sub-entities with distinct morphological features, such as Pick’s disease (PiD), corticobasal degeneration (CBD), and progressive supranuclear palsy (PSP) (Dickson et al., 2011; Kovacs, 2015). Genetically, mutations in the *MAPT* gene have been associated with FTLD-Tau (Sieben et al., 2012). FTLD-TDP is subdivided into type A-E based on the predominant type of inclusions (Mackenzie et al., 2011; Lee et al., 2017), and these have been variably associated with genetic mutations in a few different genes (e.g., *C9orf72, GRN*, *TARDBP*, *VCP*) (Sieben et al., 2012). Distinct FTLD proteinopathies cannot be differentiated during life, which poses a significant challenge for disease modifying therapies in development targeting tau and TDP-43 pathways of degeneration (Boxer et al., 2013). Thus, *postmortem* comparative studies of clinically similar FTLD-Tau and FTLD-TDP are urgently needed to improve *antemortem* diagnosis (Irwin et al., 2015).

With the increasing use of digital pathology, it is critical to develop rigorous empirically defined methods to account for the multiple pre-analytical factors that could influence these digital measurements. One major obstacle to large-scale comparative autopsy studies is the inability to simultaneously stain large amounts of tissue in a single staining batch. Yet, the use of multiple staining batches may be affected by staining batch effects, i.e., a potential important source of pre-analytical variability related to immunohistochemical (IHC) staining intensity that prevents valid inter-comparability of digital pathology measurements. It may be possible to account for this batch-related variability statistically, enabling to merge data from distinct staining batches without major issues of comparability, but we are not aware of any published methodologies used in neurodegenerative disease research.

It is advantageous for research centers to generate cumulative digital pathology data from prospective autopsies, and to build a library of digital pathology data by adding newly generated digital measurements to archived legacy data from prior autopsies. This strategy would preserve resources, and facilitate large-scale clinical, genetic and neuroimaging correlation studies urgently needed to improve the *antemortem* diagnosis of neurodegenerative diseases. While there is limited empirical evidence to guide methods for merging data obtained from tissue stained in different staining batches, this would be necessary to ameliorate comparability of digital measurements, and to prevent duplication of efforts of having to re-stain large amounts of tissue for prospective large-scale projects. Here we empirically test methodological steps to develop a working standard operating procedure (SOP) to transform digital pathology data from a new staining batch (i.e., staining batch 2 [SB2]) into equivalent values to a previous staining batch (i.e., staining batch 1 [SB1]), using a set of tissue samples stained in duplicate (i.e., in both SB1 and SB2). We test this approach in a large sample of FTLD with either tau inclusions (FTLD-Tau) or transactive response DNA binding protein 43 kDa (TDP-43) inclusions (FTLD-TDP). We focus on FTLD pathologies, since these are two distinct monoproteinopathies with varied histopathological morphologies in both grey matter (GM) and white matter (WM) (Irwin et al., 2015), and are thus ideal to test for variation due to staining batch effects as opposed to AD or LBD, which often have mixed pathology (Montine et al., 2012; McKeith et al., 2017). These data provide an important methodological approach to guide future digital pathology analysis in brain bank programs for a spectrum of age-related neurodegenerative disorders.

## Materials and Methods

### Patients

We selected a convenience sample of brain tissue from FTLD patients with high availability to use for comparative analysis of tissue samples stained in duplicate. Patients were evaluated clinically at the Penn Frontotemporal Degeneration Center or Alzheimer’s Disease Center and met clinical criteria for an FTD spectrum diagnosis (Mesulam, 2001; Rascovsky et al., 2011). Patients were autopsied at the Penn Center for Neurodegenerative Disease Research with a primary neuropathological diagnosis of FTLD (*n* = 68) with either FTLD-Tau (*n* = 26) or FTLD-TDP (*n* = 42) (Mackenzie et al., 2010; Montine et al., 2012). We did not include less common neuropathologies associated with clinical FTD, including AD, or FUS proteinopathy (Irwin et al., 2015). This study was carried out in accordance with the recommendations of the Penn Institutional Review Board (IRB) on human subjects research protections guidelines. The protocol was approved by the Penn IRB. All subjects gave written informed consent prior to participation, in accordance with the Declaration of Helsinki.

### Tissue Processing and Neuropathological Diagnosis

All tissue was processed in an identical manner as described (Toledo et al., 2014; Irwin et al., 2016b). Briefly, fresh tissue samples were fixed overnight in 10% neutral-buffered formalin, or 70% ethanol with 150 mM sodium chloride in a minority of cases (*N* = 4 in FTLD-Tau, *N* = 4 in FTLD-TDP), which has been previously validated using our digital method (Irwin et al., 2016b). Tissue samples were trimmed, placed into cassettes and processed through a series of alcohol, xylene and Surgipath EM-400 paraffin embedding media (Leica Microsystems; Buffalo Grove, IL, United States) with incubations overnight (70% ethanol × 2 h, 80% ethanol × 1 h, 95% ethanol × 1 h, 95% ethanol × 2 h, 100% ethanol × 2 h, twice, xylene × 30 min, xylene × 1 h, xylene × 1.5 h, and paraffin × 1 h, three times) in a Shandon tissue processor (Thermo Fisher Scientific; Waltham, MA, United States). All incubations were done under vacuum and at ambient temperature except paraffin (62°C). Tissue was embedded into paraffin blocks and 6-μm-thick sections were cut for analysis. For neuropathological diagnosis, tissue sections from standard brain regions were immunostained for tau, amyloid-beta, alpha-synuclein and TDP-43 using well-characterized antibodies and stained for neuritic plaques using thioflavin-S as described (Toledo et al., 2014). Neuropathological diagnoses were established by expert neuropathologists (EBL, JQT) using standard neuropathological criteria (Mackenzie et al., 2010; Montine et al., 2012; Lee et al., 2017).

For the current study, IHC was performed using well-characterized antibodies for phospho-tau (AT8; Millipore) (Mercken et al., 1992) in FTLD-Tau and TDP-43 (rat monoclonal TAR5P-1D3, p409/410; Ascenion) (Neumann et al., 2009) in FTLD-TDP. Each staining batch underwent identical processing using the same antigen retrieval methods and dilutions optimized in our lab, i.e., AT8 1:1K dilution with no antigen retrieval step; p409/410 1:500 dilution with Citrate Antigen Unmasking Solution (Vector Laboratories, Burgame, CA, United States, Catalog No: H-3300) as in previous work (Irwin et al., 2016b, 2018; Giannini et al., 2019). The same secondary antibodies were used in both staining batches of each pathology, i.e., Abcam (Cambridge, MA, United States, Goat Anti-Rat) for TDP-43 (Cat. No. ab97054) and Abcam Goat Anti-Mouse (for AT8) (Cat. No. ab97020). As a chromogen we used ImmPACT DAB kit (Vector Laboratories, Burgame, CA, United States, Cat. No. SK-4105) with VECTASTAIN ABC Kit (Vector Laboratories, Burgame, CA, United States, Cat. No. PK-4000) with identical incubation and developer times. Digital image acquisition of histology slides was performed at 20× magnification with transmitted light microscopy using Lamina (Perkin Elmer, Waltham, MA, United States) scanner, which has a slide scanning platform of 6.5 μm^2^ (i.e., pixel resolution of 0.325 μm), and camera resolution of 2560 × 2160 with a bit depth of 16. Digital image acquisition was performed using an autocorrection step for even illumination, i.e., the scanner captures 10 empty fields of view to create a compensation image used to obtain evenly illuminated composite images. Digital images were analyzed using Halo digital image software v1.90 (Indica Labs, Albuquerque, NM, United States) as described (Irwin et al., 2016b, 2018). The digital measurement performed by the Halo software uses a color deconvolution process as described in our published methods (Irwin et al., 2016b). Briefly, we used the Area Quantification v1.0 Tool in Halo to calculate the % of positive pixels from the chromogen (i.e., %AO). This tool uses color deconvolution to first separate the chromogen signal from the haematoxylin counterstain, and then it applies a minimum optical density (OD) value threshold to exclude background and count the number of positive pixels for chromogen-labeled pathology in the total ROI. Detection algorithms for pathology stain and haematoxylin counterstain were developed empirically as described (Irwin et al., 2016b). Please see Supplementary Table 1 for the specific parameters of our detection algorithms.

### Validation Procedures

We included available tissue samples from two standard autopsy-sampled regions with high availability of tissue, i.e., an anterior region such as mid-frontal cortex (MFC) and a posterior region such as angular gyrus (ANG), in which we expected a broad range of pathological severity in our FTLD cohort. We studied the relationship between duplicate measurements of digital pathology in adjacent or near adjacent sections of the same tissue block, one of which was stained in the original staining batch (SB1), and the other one in a second staining batch (SB2). To specifically assess the impact of staining batch effects, duplicate measurements of digital pathology were obtained in a nearly identical manner except for being stained in two distinct staining batches. Tissue sections were obtained from the same cutting ribbon using adjacent or semi-adjacent tissue (within ∼50 μm). By visual inspection we found no evident differences in the distribution and morphology of pathology between (semi-)adjacent slides, which were nearly identical. Using digital image analysis, we measured percent of area occupied by pathology (%AO) in regions of interest (ROIs) for both GM and WM on each section. GM ROIs were identified as the largest intact region of parallel-oriented cortex in a section of brain tissue using our previously validated sampling method (Irwin et al., 2016b). WM ROIs were sampled as the largest possible area of deep WM within a tissue section as described (Irwin et al., 2016b, 2018). To minimize sources of variation in our measurement other than staining batch effects, we used the image registration feature of the Halo software to map the ROI into equivalent regions of (semi-)adjacent tissue sections for comparable sampling between SB1 and SB2. When this was not possible, we pasted identical ROIs in a closely matched region using cellular landmarks (e.g., contours of gyri, blood vessels) to guide precision for placement.

Unusable or damaged tissue that precluded sampling in a comparable manner between adjacent sections was excluded from the analysis (*N* = 8 tissue samples in FTLD-Tau, *N* = 12 tissue samples in FTLD-TDP). Minor artifacts and vessels in brain tissue were sampled out of the area of analysis of digital images using the cropping tool in the Halo software. In total, available data from 92 tissue samples, including 39 samples from 26 patients with FTLD-Tau and 53 samples from 42 patients with FTLD-TDP, were used for this validation (see Table 1). Each tissue sample had two GM %AO measurements and two WM %AO measurements (i.e., duplicate measurements), corresponding to two nearly identical tissue sections, one stained in SB1 and the other one in SB2. Analyses were performed distinctly in FTLD-Tau and FTLD-TDP groups because these pathologies have distinct biology, morphological features and algorithms for digital image detection (Irwin et al., 2016b, 2018).

**Table 1:** Demographic and pathologic characterization of the cohort.

	FTLD-Tau (*n* = 26)	FTLD-TDP (*n* = 42)
**Available tissue**		
Total tissue samples (N)	39	53
ANG tissue samples (N)	20	38
MFC tissue samples (N)	19	15
**Demographics**		
Age at onset (y), mean ± SD	56.4 ± 12.9	59.5 ± 8.5
Age at death (y), mean ± SD	64.5 ± 13.5	65.7 ± 9.5
Disease duration (y), mean ± SD	8.6 ± 4.1	6.7 ± 4.1
Male sex, n (%)	17/26 (65.4)	21/42 (50.0)
**Autopsy**		
PMI (hr), mean ± SD	12.6 ± 6.8	12.9 ± 6.9
Brain weight (gr), mean ± SD	1089.2 ± 156.4	1106.2 ± 194.4
**Primary NPD, n (%)**		
TDP type A (incl. *GRN*)	–	17/42 (45.2)
TDP type B	–	13/42 (33.3)
TDP type C	–	7/42 (16.7)
TDP type E	–	5/42 (11.9)
CBD	5/26 (19.2)	–
PSP	4/26 (15.4)	–
PiD	9/26 (34.6)	–
Tau unclassifiable (incl. *MAPT*)	8/26 (30.8)	–
**Secondary NPD, n (%)**		
HiSc	1/26 (3.8)	5/42 (11.9)
LBD	2/26 (7.7)	1/42 (2.4)
AGD	0	2/42 (4.8)
Other^a^	0	2/42 (4.8)
**Braak^b^, n (%)**		
0	12/26 (46.2)	15/42 (35.7)
1	8/26 (30.8)	19/42 (45.2)
2	1/26 (3.8)	6/42 (14.3)
3	5/26 (19.2)	2/42 (4.8)
**CERAD, n (%)**		
0	22/26 (84.6)	29/42 (69.0)
A	3/26 (11.5)	6/42 (14.3)
B	0	5/42 (11.9)
C	1/26 (3.8)	2/42 (4.8)
**Genetic mutations, n (%)**		
*GRN*	–	8/42 (19.0)
*C9orf72*	–	15/42 (35.7)
*MAPT*	6/26 (23.1)	–


*AD, Alzheimer’s disease; AGD, argyrophilic grain disease; ANG, angular gyrus; *C9orf72*, chromosome 9 open reading frame 72; CBD, corticobasal degeneration; FTLD-Tau, frontotemporal lobar degeneration with inclusions of the tau protein; FTLD-TDP, frontotemporal lobar degeneration with inclusions of the transactive response DNA-binding protein 43 kDa; gr, grams; *GRN*, progranulin gene; hr, hours; HiSc, hippocampal sclerosis; LBD, Lewy Body disease; *MAPT*, microtubule-associated protein tau gene; MFC, mid-frontal cortex; n, number of individuals; N, number of tissue samples; NPD, neuropathological diagnosis; PiD, Pick’s disease; PMI, post-mortem interval; PSP, progressive supranuclear palsy; SD, standard deviation; TDP type A-E, FTLD-TDP subtypes (Mackenzie et al., 2011; Lee et al., 2017); y, years. ^*a*^The two individuals with other secondary pathologies had minor amounts of cerebrovascular disease in one case, and tangle-predominant senile dementia in the other case. ^*b*^For determination of Braak stages in FTLD-Tau patients, hippocampal sections were stained with an amyloid-binding dye, Thioflavin-S, to distinguish co-morbid age-related AD neurofibrillary tangle (NFT) pathology from primary FTLD-t autopathy as described (Irwin et al., 2017).*

### Statistics

All statistical analyses were performed using R Statistical Software 3.4.1. Since %AO measurements were not normally distributed, we applied natural log (ln) transformation and confirmed normal distribution graphically. We used ln-transformed data (i.e., ln %AO) in all our validation analyses. Digital pathology measurements were validated through comparison to gold-standard ordinal ratings (Supplementary Figure 1) as previously done (Irwin et al., 2016b, 2018). In this validation dataset (FTLD-Tau = 39, FTLD-TDP = 53; Table 1), all tissue samples were stained in duplicate in SB1 and SB2, which gave us the chance (1) to determine the impact of staining batch effects in a large sample of data, and (2) to assess our proposed transformation method using a planned train-test set design.

First, to determine the impact of staining batch effects, duplicate measurements of pathology in GM and WM ROIs from SB1 and SB2 were compared using the Bland-Altman (BA) statistics to test the mean difference between staining batches. We tested the null hypothesis that the mean difference between SB1 and SB2 measurements equaled zero using a one-sided *t*-test. Significant results were interpreted as providing evidence for a difference between these duplicate measurements (Bland and Altman, 1986). Subsequently, the relationship between duplicate measurements of pathology was explored using univariate linear regression. Linear modeling used SB1 measurements as dependent variable and SB2 measurements as independent variable. Based on the strong linear relationship in both FTLD-Tau and FTLD-TDP, we proposed to use the linear equivalence equation to transform SB2 data into values equivalent to SB1: *transformed SB2 (t-SB2) = beta ^∗^ SB2 + intercept.*

**FIGURE 1:**
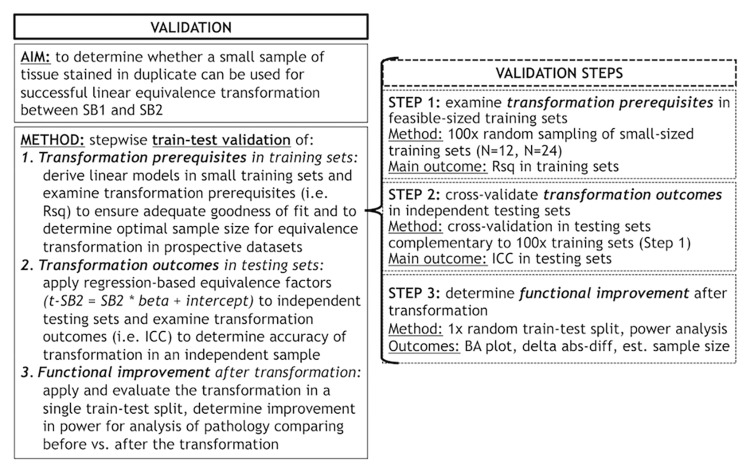
Objectives and methods of this validation study and stepwise validation protocol. Panel outlines the aim and methods of our validation study to account for staining batch effects in digital pathology, including a stepwise protocol to assess relevant aspects of our proposed methodology. delta abs-diff, change in absolute difference; est. sample size, estimated required sample size in a power analysis; ICC, intraclass correlation coefficient; N, number of tissue samples; Rsq, R squared; SB1, staining batch 1 (original); SB2, staining batch 2 (new); t-SB2, transformed staining batch 2 (new).

Second, we used a planned train-test set design (see below for details) to validate our proposed transformation method, which relies upon the use of a small set of tissue stained in each prospective staining run to account for batch effects in digital measurements. We validated this method using a validation protocol involving different steps (Figure 1) to empirically determine the optimal conditions for a successful transformation of SB2 measurements into SB1-equivalent units (i.e., t-SB2). We first looked at transformation prerequisites (i.e., consistent and sufficiently high goodness of fit in linear models) in randomly assembled training sets using a relatively small sample size (i.e., *N* = 12–24) feasible for use in prospective staining runs (i.e., one-half to one full staining-rack in our lab) (Step 1). Thereafter, we applied regression-based equivalence factors (i.e., beta, intercept) to complementary independent testing sets. Next, we cross-validated transformation outcomes in these testing sets (Step 2) to verify the accuracy of transformation (i.e., whether t-SB2 values approximated SB1 values). We used Step 1 and Step 2 to determine whether a relatively small set of control tissue (*N* = 12–24) could be used in our SOP for prospective data addition to existing datasets. Finally, we looked at functional outcomes of this approach to facilitate and improve future studies, such as the reduction in batch-related difference in duplicate measurements and the increase in statistical power using transformed %AO values as opposed to untransformed values from tissue stained in different batches (Step 3).

#### Step 1: Examine Transformation Prerequisites in Feasible-Sized Training Sets

Our first analysis was to determine feasibility of using a small set of control tissue stained in each prospective staining run by testing whether small training sets (i.e., *N* = 12–24) could provide adequate transformation prerequisites for our transformation method. We performed linear regressions relating SB1 (dependent variable) to SB2 (independent variable) data in randomly subsampled training sets of *N* = 12 and *N* = 24 sample size. We performed 100 iterations per training-set sample size, and we obtained mean, standard deviation and a non-parametric quantile-based 95% confidence interval (CI) for Rsq, beta and intercept. Mean Rsq was our main outcome as a measure of the goodness of fit of linear modeling in these random iterations.

#### Step 2: Cross-Validate Transformation Outcomes in Independent Testing Sets

Next, we applied equivalence factors (i.e., beta, intercept) of the linear equivalence equation from iterated linear models (Step 1) to independent testing sets for cross-validation. Each *N* = 12 and *N* = 24 training set was retested on the respective complementary testing set (FTLD-Tau: train = 24/test = 15 or train = 12/test = 27; FTLD-TDP: train = 24/test = 29 or train = 12/test = 41). Our main transformation outcome was intraclass correlation coefficient (ICC) to assess equivalence between transformed SB2 measurements (i.e., t-SB2) and original measurements from SB1. We defined an optimal transformation as ICC ≥ 0.8 and determined the frequency of optimal transformations out of 100 iterations per training-set sample size (100×*N* = 24 training sets, 100×*N* = 12 training sets) in GM and WM in both FTLD-Tau and FTLD-TDP.

#### Step 3: Determine Functional Improvement After Transformation

Finally, we were interested in determining whether the application of our transformation method resulted in improved functional outcomes for the performance of digital pathology analysis. To this end, we used a single random train-test split using a *N* = 12 training set in FTLD-Tau, and a *N* = 24 training set in FTLD-TDP, and we applied the transformation to independent testing sets including all remaining data in FTLD-Tau (*N* = 27) and FTLD-TDP (*N* = 29). Here, we assessed the impact of the transformation by testing whether there was a reduction in the difference between duplicate measurements from different staining batches. We estimated the mean difference between duplicate measurements, and visually compared Bland-Altman plots of test-retest agreement before and after the transformation (Bland and Altman, 1986). Additionally, we estimated the change in absolute difference in measurements (i.e., delta abs-diff) between after the transformation (i.e., absolute difference between t-SB2 and SB1) and before the transformation (i.e., absolute difference between SB2 and SB1). We tested whether delta abs-diff equaled zero using a one-sample *t*-test, where a significant finding (*p* < 0.05) indicated a significant reduction in batch-related difference in measurements after applying the transformation.

Finally, we performed a proof-of-concept power analysis to see how much increased power could be obtained using alternatively (1) data merged from a random selection of the original staining batch and the new staining batch without transformation (i.e., merged untransformed = SB1 + SB2), and (2) data merged from a random selection of the original staining batch and the new staining batch after transformation (i.e., merged transformed = SB1 + t-SB2). To this end, we used data from the MFC region to derive linear models in both FTLD-Tau (*N* = 19) and FTLD-TDP (*N* = 15). Next, we applied the transformation to independent testing sets with data exclusively from the ANG region (FTLD-Tau = 20; FTLD-TDP = 38). Merged untransformed (SB1 + SB2) and merged transformed (SB1 + t-SB2) variables were obtained in testing sets through random assignment with a 50:50 ration between SB1 and SB2/t-SB2. For our proof-of-concept power analysis, we calculated the standard deviation in these two sets of data (i.e., merged untransformed, merged transformed) and we used it as an approximation of the overall variance (ANG vs. any hypothetical region) for possible regional comparisons. We estimated the sample size necessary to detect varying differences between mean ANG and another hypothetical regional mean, i.e., 0.2, 0.5, and 0.8, corresponding to small, medium and large effect sizes (Cohen, 1988). The power analysis was performed with power of 0.8 and alpha of 0.05.

#### Analysis of Generalizability: Replication of Validation Outcomes Using an Open-Source Digital Platform

To test the generalizability of our approach, we replicated the main analyses of this validation using an open-source image analysis tool, i.e., QuPath (Bankhead et al., 2017). In QuPath, we quantified %AO by pathology importing the same RGB color deconvolution algorithms derived in Halo for tau and TDP-43 inclusions (Supplementary Table 1) in matched ROIs using the same cellular landmarks for precise ROI placement in QuPath as in Halo. First, we compared %AO measurements between SB1 and SB2 in comparable ROIs to assess whether similar staining batch effects were observable in another digital platform. Next, we applied the transformation method to verify the accuracy of transformation in data obtained from this open-source platform. Our main outcome measures were ICC, delta abs-diff and Bland-Altman statistics after transformation in a single random train-test split (Step 3).

Finally, we also tested an alternative approach to transformation of %AO values to correct for staining batch effects using the “Estimate stain vectors” tool in QuPath (Macenko et al., 2009), which enables to empirically and systematically develop a new color deconvolution algorithm for both haematoxylin counterstain and DAB chromogen in a subsequent staining batch. This QuPath function detects RGB color signal and plots individual pixel signal in each vector of RGB (stain vector plots), where the accuracy of color deconvolution is defined by the presence of pixels within the confines of the stain vector plots. To develop optimized algorithms using this tool, we used the same approach as the one we used to develop our original algorithms as published (Irwin et al., 2016b). Briefly, RGB and minimum OD values are estimated empirically in five random slides. Next, the final RGB and minimum OD parameters of the optimized algorithms are calculated as the average from these random slides. In this supplementary analysis, we derived optimized algorithms in SB2 to compare optimized SB2 measurements to original SB1 measurements analyzed in QuPath. We tested agreement between the original algorithm in SB1 and the optimized SB2 algorithm in the full dataset using Bland-Altman analysis for test-retest agreement.

## Results

### Data Comparison Between Staining Batches

Patient demographics are summarized in Table 1. Consistent with our previous validation of specific algorithms for digital histopathological analysis, we found digital %AO measurements reflected gold-standard ordinal ratings of pathology (Supplementary Figure 1).

**FIGURE 2:**
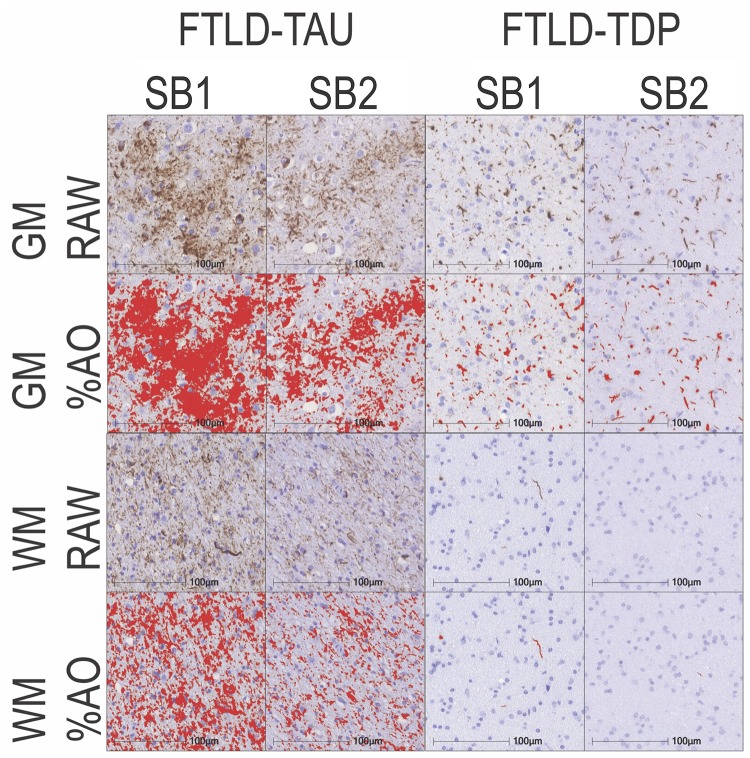
Representative photomicrographs of staining batch variability in FTLD-Tau and FTLD-TDP. Photomicrographs depict a mid-frontal cortex section of FTLD-Tau (Corticobasal degeneration; left) and FTLD-TDP (TDP type A; right) with raw and digital %AO detection red overlay of pathology in gray matter (top) and white matter (bottom) in approximate matched areas in staining batch 1 (SB1) vs. staining batch 2 (SB2). There is slightly darker DAB chromogen signal and thus greater %AO in SB1 compared to SB2. Scale bar = 100 μm. FTLD-Tau, frontotemporal lobar degeneration with inclusions of the tau protein; FTLD-TDP, frontotemporal lobar degeneration with inclusions of the transactive response DNA-binding protein 43 kDa; GM, gray matter; SB1, staining batch 1 (original); SB2, staining run 2 (new); WM, white matter.

**FIGURE 3:**
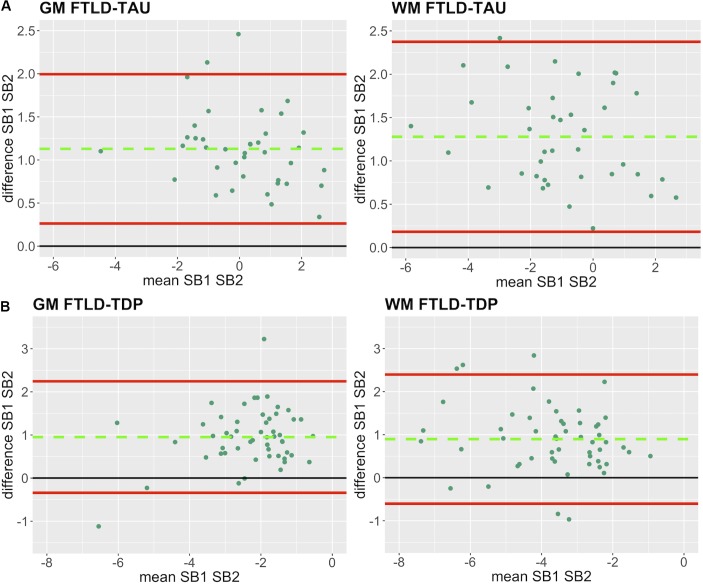
Bland-Altman plots of test-retest agreement between duplicate measurements of pathology from two distinct staining batches. Bland-Altman plots show test-retest agreement between SB1 and SB2 measurements of digital pathology (i.e., ln %AO). The green dashed line indicates the mean difference between SB1 and SB2 measurements, while the red solid lines mark the 95% limits of agreement between the two measurements. We find that mean difference between SB1 and SB2 significantly differs from zero (*p* < 0.001) in FTLD-Tau **(A)** and FTLD-TDP **(B)** in both GM and WM. FTLD-Tau, frontotemporal lobar degeneration with inclusions of the tau protein; FTLD-TDP, frontotemporal lobar degeneration with inclusions of the transactive response DNA-binding protein 43 kDa; GM, gray matter; SB1, staining batch 1 (original); SB2, staining batch 2 (new); WM, white matter.

**FIGURE 4:**
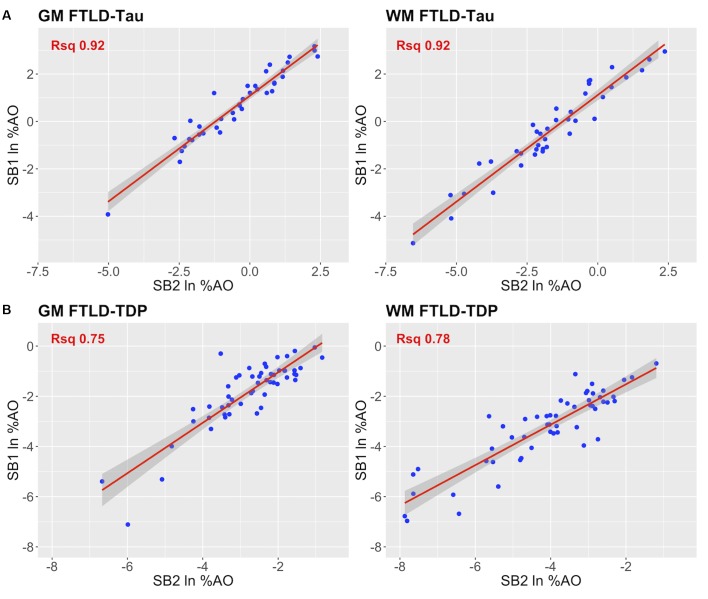
Linear relationship between duplicate measurements of pathology from two distinct staining batches (SB1 *Y*-axis, SB2 *X*-axis). Scatterplots display the linear relationship between duplicate measurements of digital pathology (i.e., ln %AO) from SB1 (*y*-axis) and SB2 (*x*-axis) in FTLD-Tau **(A)** and FTLD-TDP **(B)**, for both GM and WM measurements. In FTLD-Tau GM, the model Rsq is 0.92 (*p* < 0.001); in FTLD-Tau WM, the model Rsq is 0.92 (*p* < 0.001). In FTLD-TDP GM, the model Rsq is 0.75 (*p* < 0.001); in FTLD-TDP WM, the model Rsq is 0.78 (*p* < 0.001). FTLD-Tau, frontotemporal lobar degeneration with inclusions of the tau protein; FTLD-TDP, frontotemporal lobar degeneration with inclusions of the transactive response DNA-binding protein 43 kDa; GM, gray matter; ln %AO, natural logarithmic transformation of percent area occupied by pathology; Rsq, R squared; SB1, staining batch 1 (original); SB2, staining batch 2 (new); WM, white matter.

In our analysis of the impact of staining batch effects on digital measurements (see Figure 2 for a visual representation of %AO by tau or TDP-43), we found that in FTLD-Tau the mean difference between SB1 and SB2 duplicate measurements was 1.13 ± 0.44 in GM and 1.28 ± 0.56 in WM. In FTLD-TDP, the mean difference between SB1 and SB2 duplicate measurements was 0.95 ± 0.66 in GM and 0.90 ± 0.77 in WM. Bland-Altman statistics showed that the mean difference between duplicate measurements significantly differed from zero (one-sided *t*-test, *p* < 0.001) in both GM and WM in FTLD-Tau and FTLD-TDP (Figure 3), suggesting that %AO measurements from different staining batches were not equivalent. The relationship between SB1 and SB2 was further explored using univariate linear regression, where SB1 data was employed as the dependent variable and SB2 as the independent variable. All models (i.e., GM and WM in FTLD-Tau and FTLD-TDP) were highly significant, indicating a linear relationship between duplicate measurements from our two staining batches (Figure 4). In FTLD-Tau, both GM and WM models had Rsq of 0.92; in FTLD-TDP, the Rsq was 0.75 in GM and 0.78 in WM. All summary statistics for SB1 and SB2 data are displayed in Supplementary Table 2.

### Step 1: Examine Transformation Prerequisites in Feasible-Sized Training Sets

Since it is not practical to use a large number of duplicate tissue samples for each prospective staining batch in all future large-scale studies, we aimed to first determine whether a small set of tissue stained in duplicate could suffice to obtain an accurate transformation. We used samples of *N* = 12 and *N* = 24 as our training sets, i.e., half- or one-full rack in our staining batches. We performed iterations (100×) of linear modeling in training sets, looking at Rsq values as transformation prerequisites, and beta and intercept as equivalence factors (Table 2). In FTLD-Tau GM, the mean Rsq was 0.92 ± 0.03 in *N* = 24 sets and 0.91 ± 0.05 in *N* = 12 sets; the models were significant (*p* < 0.05) in 100% of *N* = 24 iterations and *N* = 12 iterations. In FTLD-Tau WM, the mean Rsq was 0.91 ± 0.02 in *N* = 24 sets and 0.90 ± 0.06 in *N* = 12 sets; the models were significant (*p* < 0.05) in 100% of *N* = 24 iterations and *N* = 12 iterations. In FTLD-TDP GM, the mean Rsq was 0.76 ± 0.10 in *N* = 24 sets and 0.72 ± 0.18 in *N* = 12 sets; the models were significant (*p* < 0.05) in 100% of *N* = 24 iterations and 97% of *N* = 12 iterations. In TDP WM, the mean Rsq was 0.78 ± 0.07 in *N* = 24 sets and 0.76 ± 0.12 in *N* = 12 sets; the models were significant (*p* < 0.05) in 100% of *N* = 24 iterations and 100% of *N* = 12 iterations. Overall we found a consistent strong linear association between %AO measurements from different staining batches equally in *N* = 24 and *N* = 12 training sets in FTLD-Tau, while FTLD-TDP had greatest reliability of this association in training sets of *N* = 24 sample size (Table 2).

**Table 2:** Transformation prerequisites and equivalence factors from iterated (×100) linear regression in feasible-sized training sets (Step 1).

	Size (N)	Iterations	Rsq mean	Rsq SD	Rsq 2.5Q–97.5Q		Beta mean	Beta SD	Beta 2.5Q–97.5Q		Itc mean	Itc SD	Itc 2.5Q–97.5Q	
FTLD-Tau GM	Tot	1	0.92	–	–	–	0.89	–	–	–	1.08	–	–	–
	24	100	0.92	0.03	0.87	0.96	0.89	0.04	0.80	0.96	1.08	0.04	0.99	1.16
	12	100	0.91	0.05	0.79	0.98	0.89	0.07	0.74	1.01	1.09	0.11	0.88	1.31
FTLD-Tau WM	Tot	1	0.92	–	–	–	0.90	–	–	–	1.11	–	–	–
	24	100	0.91	0.02	0.88	0.94	0.90	0.03	0.84	0.96	1.12	0.09	0.93	1.25
	12	100	0.90	0.06	0.77	0.97	0.90	0.08	0.75	1.05	1.12	0.19	0.79	1.49
FTLD-TDP GM	Tot	1	0.75	–	–	–	1.00	–	–	–	0.96	–	–	–
	24	100	0.76	0.10	0.44	0.87	1.00	0.14	0.74	1.25	0.95	0.35	0.37	1.67
	12	100	0.72	0.18	0.26	0.94	0.98	0.22	0.57	1.41	0.90	0.55	-0.12	1.98
FTLD-TDP WM	Tot	1	0.78	–	–	v	0.81	–	–	–	0.09	–	–	–
	24	100	0.78	0.07	0.64	0.87	0.81	0.07	0.65	0.93	0.10	0.29	-0.56	0.57
	12	100	0.76	0.12	0.47	0.92	0.81	0.13	0.61	1.10	0.14	0.53	-0.66	1.33


*FTLD-Tau, frontotemporal lobar degeneration with inclusions of the tau protein; FTLD-TDP, frontotemporal lobar degeneration with inclusions of the transactive response DNA-binding protein 43 kDa; GM, gray matter; Itc, intercept; N, number of tissue samples; Q, quantile; Rsq, R squared; SD, standard deviation; Tot, total dataset; WM, white matter. Table shows transformation prerequisites (i.e., Rsq) and equivalence factors (i.e., beta, intercept) in randomly subsampled training sets of small sample size, corresponding to a half (*N* = 12) or one full rack (*N* = 24) in staining batches, feasible for use in prospective transformations. We performed 100 iterations of the linear regression, and we report mean, standard deviation and a non-parametric quantile-based confidence interval (2.5–97.5% of the distribution) for R squared, beta and intercept values of the linear models. For comparison, we also show these parameters from linear models obtained in the total datasets (i.e., FTLD-Tau GM/WM, FTLD-TDP GM/WM).*

### Step 2: Cross-Validate Transformation Outcomes in Independent Testing Sets

Next, we cross-validated equivalence factors derived in Step 1 on independent testing sets including all remaining tissue samples not used in the training set (Table 3). We were interested in comparing transformation outcomes resulting from the application of equivalence factors from *N* = 12 as opposed to *N* = 24 training sets. We looked at the ICC as main transformation outcome and we set a value of ≥0.8 as our threshold for an optimal transformation. In FTLD-Tau GM, *N* = 24 training sets resulted in a mean ICC of 0.95 ± 0.02 in testing sets, while *N* = 12 training sets resulted in a mean ICC of 0.95 ± 0.01. Similarly, in FTLD-Tau WM, *N* = 24 training sets resulted in a mean ICC of 0.95 ± 0.02 in testing sets, while *N* = 12 training sets resulted in a mean ICC of 0.95 ± 0.01. We obtained optimal transformation outcomes in 100% of transformations in both GM and WM in FTLD-Tau (Table 3). In FTLD-TDP GM, *N* = 24 training sets resulted in a mean ICC of 0.82 ± 0.05 in testing sets, while *N* = 12 training sets resulted in a mean ICC of 0.81 ± 0.06. We found optimal transformation outcomes in 72% of transformations using *N* = 24 training sets and 70% using *N* = 12 training sets, while most remaining transformations (i.e., 25% using *N* = 24 training sets and 26% using *N* = 12 training sets) resulted in a moderate ICC between 0.7 and 0.8. In FTLD-WM, *N* = 24 training sets resulted in a mean ICC of 0.86 ± 0.03 in testing sets, while *N* = 12 training sets resulted in a mean ICC of 0.85 ± 0.03. We obtained optimal transformation outcomes in 98% of transformations using *N* = 24 training sets and 95% using *N* = 12 training sets (Table 3). Based on these frequencies, in prospective analyses we propose to use at least *N* = 12 training sets for FTLD-Tau and *N* = 24 training sets for FTLD-TDP, where we find the best compromise between feasibility of use and reliability of outcomes.

**Table 3:** Transformation outcomes in independent complementary testing sets (Step 2).

	Training sets (Step 1)		Independent testing sets (Step 2)					
	Size (N)	Iterations	Size (N)	ICC mean	ICC SD	ICC 2.5Q–97.5Q		ICC ≥ 0.8 (%)
FTLD-Tau GM	24	100	15	0.95	0.02	0.90	0.98	100
	12	100	27	0.95	0.01	0.92	0.97	100
FTLD-Tau WM	24	100	15	0.95	0.02	0.91	0.98	100
	12	100	27	0.95	0.01	0.92	0.96	100
FTLD-TDP GM	24	100	29	0.82	0.05	0.69	0.91	72
	12	100	41	0.81	0.06	0.69	0.89	70
FTLD-TDP WM	24	100	29	0.86	0.03	0.80	0.91	98
	12	100	41	0.85	0.03	0.78	0.89	95


*FTLD-Tau, frontotemporal lobar degeneration with inclusions of the tau protein; FTLD-TDP, frontotemporal lobar degeneration with inclusions of the transactive response DNA-binding protein 43 kDa; ICC, intraclass correlation coefficient; GM, gray matter; N, number of tissue samples; Q, quantile; SD, standard deviation; WM, white matter. We performed 100 iterations of linear regression in training sets of *N* = 12 and *N* = 24 sample size, and applied equivalence factors to independent testing sets including all remaining tissue samples in each iteration. Here, we report transformation outcomes (i.e., ICC) in these complementary testing sets. We report mean, standard deviation and a non-parametric quantile-based confidence interval (2.5–97.5% of the distribution) for the ICC. Additionally, we report the frequency of optimal transformations out of 100 iterations per group based on our threshold of ICC ≥ 0.8.*

### Step 3: Determine Functional Improvement After Transformation

**FIGURE 5:**
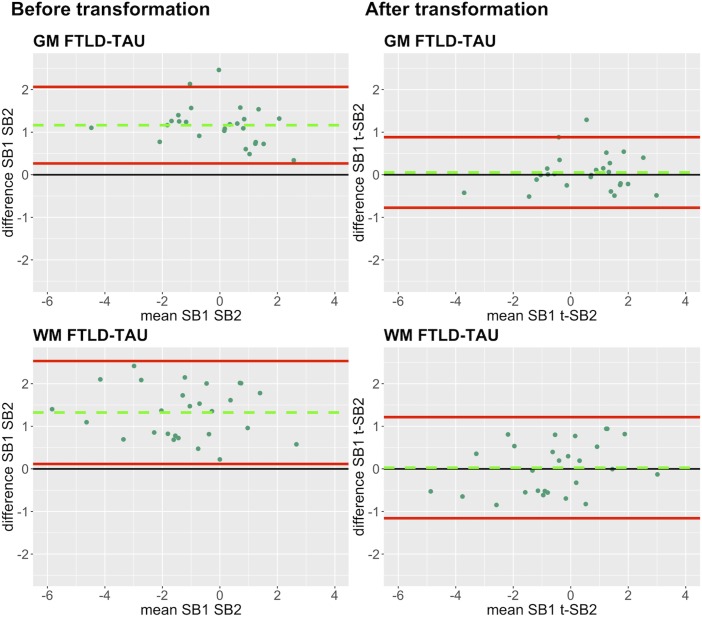
Bland-Altman plots of test-retest agreement between duplicate measurements of pathology before vs. after transformation in FTLD-Tau. Plots portray test-retest agreement between duplicate measurements of digital pathology (i.e., ln %AO) in FTLD-Tau from SB1 and SB2 before and after transforming the data using our validated linear regression-based method. Here we illustrate the reduction in batch-related difference in digital measurements resulting from the application of our transformation method in a single train-test split in FTLD-Tau (Step 3). The green dashed line indicates the mean difference between SB1 and SB2 measurements, while the red solid lines mark the 95% limits of agreement between the two measurements. We find that mean difference between SB1 and SB2/t-SB2 is significantly different from zero before transformation (*p* < 0.05, one-sample *t*-test), whereas it is not significantly different from zero after transformation (*p* > 0.05) in both GM and WM. FTLD-Tau, frontotemporal lobar degeneration with inclusions of the tau protein; GM, gray matter; SB1, staining batch 1 (original); SB2, staining batch 2 (new); t-SB2, transformed staining batch 2 (new); WM, white matter.

**FIGURE 6:**
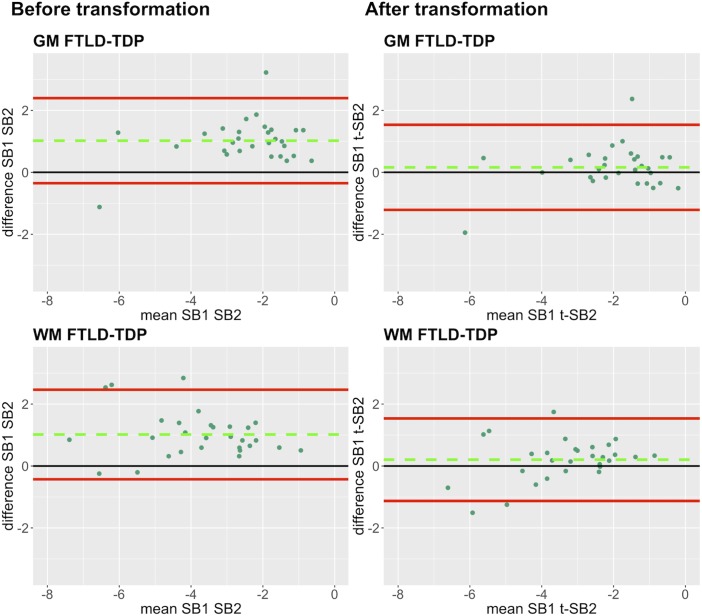
Bland-Altman plots of test-retest agreement between duplicate measurements of pathology before vs. after transformation in FTLD-TDP. Plots portray test-retest agreement between duplicate measurements of digital pathology (i.e., ln %AO) in FTLD-TDP from SB1 and SB2 before and after transforming the data using our validated linear regression-based method. Here we illustrate the reduction in batch-related difference in digital measurements resulting from the application of our transformation method in a single train-test split in FTLD-TDP (Step 3). The green dashed line indicates the mean difference between SB1 and SB2 measurements, while the red solid lines mark the 95% limits of agreement between the two measurements. We find that mean difference between SB1 and SB2/t-SB2 is significantly different from zero before transformation (*p* < 0.05, one-sample *t*-test), whereas it is not significantly different from zero after transformation (*p* > 0.05) in both GM and WM. FTLD-TDP, frontotemporal lobar degeneration with inclusions of the transactive response DNA-binding protein 43; GM, gray matter; SB1, staining batch 1 (original); SB2, staining batch 2 (new); t-SB2, transformed staining batch 2 (new); WM, white matter.

**FIGURE 7:**
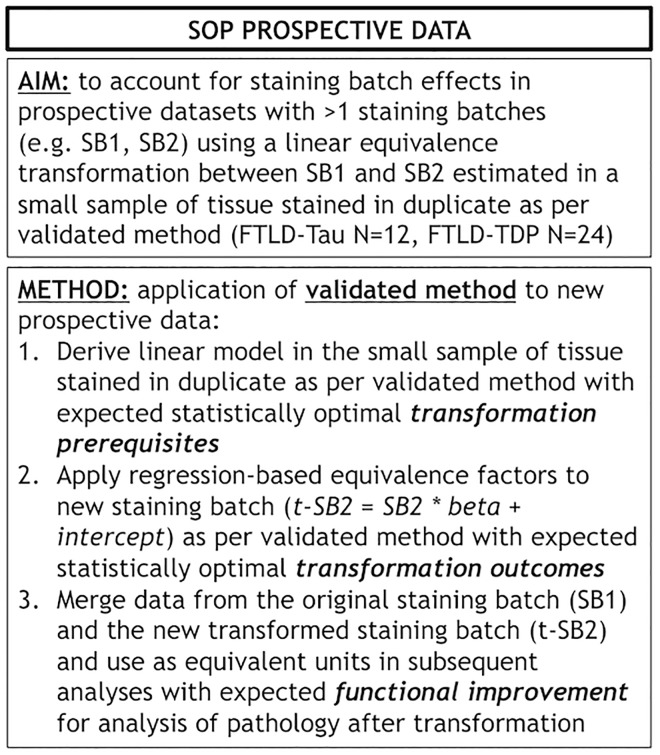
Standard operating procedure for prospective use of our validated transformation method. Panel outlines a standard operating procedure (SOP) for prospective addition of new data to existing datasets where we use our validated transformation method to account for staining batch effects. FTLD-Tau, frontotemporal lobar degeneration with inclusions of the tau protein; FTLD-TDP, frontotemporal lobar degeneration with inclusions of the transactive response DNA-binding protein 43; LMN, number of tissue samples; SB1, staining batch 1 (original); SB2, staining batch 2 (new); SOP, standard operating procedure; t-SB2, transformed staining batch 2 (new).

Finally, we applied our cross-validated method to a single, randomly obtained train-test split to determine the improvement in functional outcomes, such as the reduction in batch-related difference between digital measurements. We checked the reliability of our transformation method as in the prior steps, by looking at transformation prerequisites in training sets (FTLD-Tau = 12, FTLD-TDP = 24) and transformation outcomes in testing sets (FTLD-Tau = 27, FTLD-TDP = 29) (Table 4). In FTLD-Tau, training sets had an Rsq of 0.92 in GM and 0.97 in WM. In complementary testing sets, ICC was 0.96 in GM and 0.95 in WM. Before transformation, Bland-Altman statistics showed highly significant mean difference between duplicate %AO measurements in both GM and WM. After transformation, we found a significant reduction in absolute difference (i.e., delta abs-diff) in both GM (*p* < 0.001) and WM (*p* < 0.001) %AO to a mean difference that approached zero, suggesting improved test-retest agreement (Figure 5). In FTLD-TDP, training sets had an Rsq of 0.70 in GM and of 0.75 in WM. In complementary testing sets, ICC was 0.88 in both GM and WM. While before transformation, the mean difference between duplicate %AO measurements was highly significant using Bland-Altman statistics, after transformation there was a significant reduction in absolute difference (i.e., delta abs-diff) in both GM (*p* < 0.001) and WM (*p* < 0.001) %AO to a mean difference that approached zero, similarly suggesting improved test-retest agreement (Figure 6). These findings help us validate the functional implications of our SOP, where we propose to use a small sample of tissue stained in each prospective staining batch to transform newly acquired data into values equivalent to previously generated data, thereby accounting for staining batch effects (Figure 7).

**Table 4:** Application of transformation method in a single train-test split to determine reduction in batch-related difference in measurements (Step 3).

	Train (N)	Rsq	Beta	Itc	Test (N)	ICC	Mean diff before	BA before sig.	Mean diff after	BA after sig.	Delta abs- diff	Delta abs-diff sig.
**1× train-test split FTLD-Tau**					
GM	12	0.92	0.91	1.05	27	0.96	–1.16	5.04e-13	–0.05	0.513	–0.86	2.89e-11
WM	12	0.97	0.86	1.03	27	0.95	–1.32	2.08e-11	–0.03	0.808	–0.79	4.92e-07
**1× train-test split FTLD-TDP**					
GM	24	0.70	1.01	0.89	29	0.88	–1.02	1.48e-08	–0.16	0.226	–0.61	1.92e-08
WM	24	0.75	0.79	–0.08	29	0.88	–1.02	4.37e-08	–0.20	0.117	–0.50	1.31e-04


*BA, Bland-Altman statistics; Delta abs-diff, change in absolute difference; diff, difference; FTLD-Tau, frontotemporal lobar degeneration with inclusions of the tau protein; FTLD-TDP, frontotemporal lobar degeneration with inclusions of the transactive response DNA-binding protein 43 kDa; ICC, intraclass correlation coefficient; Itc, intercept; GM, gray matter; N, number of tissue samples; Rsq, R squared; sig., significance; WM, white matter. Here, we display the results of the application of our cross-validated transformation method in a single randomly obtained train-test split. On the left side, table shows transformation prerequisites (i.e., Rsq) and equivalence factors (i.e., beta, intercept) in training sets (GM/WM in FTLD-Tau and FTLD-TDP). On the right side, we report corresponding transformation outcomes (i.e., ICC) in the complementary testing sets. Additionally, we look at measures of test-retest agreement, i.e., mean difference between SB1 and SB2 before and after the transformation, with Bland-Altman statistics (*p* < 0.05, significant mean difference between SB1 and SB2/t-SB2; *p* > 0.05, non-significant mean difference between SB1 and SB2/t-SB2), as well as the decrease in absolute difference tested with a one-sample *t*-test statistics (*p* < 0.05, significant reduction in difference between duplicate measurements after the transformation).*

Finally, we performed a proof-of-concept power analysis to determine the magnitude of improved statistical power after the application of our transformation method, compared to a standard approach using datasets of untransformed %AO obtained from two distinct staining batches. We compared the use of merged untransformed (SB1 + SB2) and merge transformed (SB1 + t-SB2) data in tissue from ANG (FTLD-Tau = 20, FTLD-TDP = 38), and we estimated the necessary sample size to detect a small, medium or large effect size when compared to another hypothetical brain region (power = 0.8, alpha = 0.05). We found that the application of our transformation method resulted in a reduction in estimated sample size required for analysis, i.e., -40% in FTLD-Tau GM, -34% in FTLD-Tau WM, -20% in FTLD-TDP GM, and -30% in FTLD-TDP WM (Table 5).

**Table 5:** Outcomes of power analysis using merged SB1 and SB2/t-SB2 data to measure improvement before vs. after the transformation (Step 3).

	Merged untransformed (SB1 + SB2)			Merged transformed (SB1 + t-SB2)			Percent reduction
	ANG SD	Effect size	Est. sample	ANG SD	Effect size	Est. sample	Est. sample (%)
FTLD-Tau GM	1.96	0.8	95	1.52	0.8	58	–39
(*N* = 20)	1.96	0.5	242	1.52	0.5	146	–40
	1.96	0.2	1505	1.52	0.2	906	–40
FTLD-Tau WM	2.17	0.8	116	1.77	0.8	77	–33
(*N* = 20)	2.17	0.5	296	1.77	0.5	197	–34
	2.17	0.2	1845	1.77	0.2	1224	–34
FTLD-TDP GM	1.35	0.8	45	1.20	0.8	36	–20
(*N* = 38)	1.35	0.5	115	1.20	0.5	92	–20
	1.35	0.2	713	1.20	0.2	567	–20
FTLD-TDP WM	1.55	0.8	60	1.30	0.8	42	–29
(*N* = 38)	1.55	0.5	152	1.30	0.5	107	–30
	1.55	0.2	943	1.30	0.2	661	–30


*ANG, angular gyrus; Est., estimated; FTLD-Tau, frontotemporal lobar degeneration with inclusions of the tau protein; FTLD-TDP, frontotemporal lobar degeneration with inclusions of the transactive response DNA-binding protein 43 kDa; GM, gray matter; N, number of tissue samples; SB1, original staining batch; SB2, new staining batch (untransformed); SD, standard deviation; t-SB2, new staining batch (transformed); WM, white matter. Here, we show a power analysis to estimate the sample size necessary for an independent samples *t*-test testing pathology burden (i.e., mean ln %AO) in ANG against any other hypothetical brain region. Our aim was to measure the improvement in power after transformation by comparing (1) data merged from the original staining batch and the new staining batch without transformation (i.e., merged untransformed = SB1 + SB2), and (2) data merged from the original staining batch and the new staining batch after transformation (i.e., merged transformed = SB1 + t-SB2). We calculated ANG SD in these two sets of data and used it as an approximation of the overall variance (ANG vs. hypothetical region). We used effect sizes of 0.2, 0.5, and 0.8, corresponding to small, medium, and large effect sizes (Cohen, 1988), to estimate the sample size necessary (i.e., Est. sample) to detect a difference between mean ANG and another hypothetical regional mean. Each power analysis used alpha 0.05 and power 0.8. We measured the change between merged untransformed and merged transformed data by means of a percent reduction in estimated sample size.*

### Exploratory Analysis: Application of Validated Method to Other Sources of Pre-analytical Variability

We tested whether this method could help account for other sources of pre-analytical variability. In our brain bank, tissue that is not sampled for IHC analysis is frozen at -80 degrees for use in biochemical studies (Toledo et al., 2014). It would be advantageous to harvest frozen tissue in key regions that are not routinely sampled by traditional protocols (Montine et al., 2012) for more extensive regional and bilateral analyses in FTD (Irwin et al., 2018; Giannini et al., 2019). To test this approach, we compared digital %AO measurements between standard fresh tissue fixed at autopsy and legacy frozen tissue samples. We obtained tissue samples from frozen cortical slabs adjacent to those sampled fresh at autopsy (*N* = 16 in FTLD-Tau, *N* = 12 in FTLD-TDP), and allowed the frozen samples to thaw prior to fixation overnight in 10% neutral buffered formalin. All subsequent processing was done in an identical manner to standard samples obtained fresh at autopsy (Toledo et al., 2014). Frozen-fixed and standard (i.e., fresh-fixed) tissue samples from adjacent cortical slabs in the MFC were stained in the same staining batch, and %AO was measured using our standard digital image approach (please see methods). We performed equivalence analyses and found a significant linear association between frozen-fixed and fresh-fixed duplicate measurements in both FTLD-Tau (Rsq = 0.77, *p* < 0.001) and FTLD-TDP (Rsq = 0.70, *p* < 0.001) in the MFC region (Supplementary Figure 2), suggesting that it may be possible to use a similar SOP approach to the one we propose for staining batch effects (Figure 7) to account for other pre-analytical factors, such as processing of frozen tissue for IHC analysis.

### Analysis of Generalizability: Replication of Validation Outcomes Using an Open-Source Digital Platform

We examined whether our findings of staining batch variability and improved agreement after transformation were reproducible using another digital histopathology platform, i.e., QuPath (Bankhead et al., 2017), by importing our color deconvolution algorithm parameters (Supplementary Table 1) in this software and performing identical analyses. First, we found that %AO measurements obtained from identical images in Halo and QuPath platforms were highly correlated for both FTLD-Tau and FTLD-TDP (Rsq ≥ 0.84, *p* < 0.001; Supplementary Figure 3), suggesting a strong association of %AO measurements of pathology across platforms. Next, we compared duplicate measurements between staining batches in the total FTLD-Tau and FTLD-TDP datasets (Supplementary Table 3), and found a significant difference between SB1 and SB2 QuPath measurements (*p* < 0.001 all), which were linearly related (*p* < 0.001 all) similar to our analyses above (Figure 3, 4 and Table 2). Application of the transformation method to QuPath data enabled to account for this variability as in Step 3 (Supplementary Table 4); Bland-Altman analysis showed improved test-retest agreement after transformation in both FTLD-Tau and FTLD-TDP (Supplementary Figures 4, 5) similar to our analyses using the Halo platform (Table 4 and Figure 5, 6).

Finally, we performed an exploratory analysis to test the ability of an empiric stain detection algorithm approach to account for staining batch effects of both haematoxylin and DAB in SB2. We used the “Estimate stain vectors” function in QuPath to define optimum RGB values for DAB and haematoxylin in SB2, resulting in optimized detection algorithms for SB2 (Supplementary Table 5). We thus compared the optimized SB2 measurements to original SB1 measurements in QuPath in the total dataset (see section “Materials and Methods”). Using this approach, we found good test-retest agreement between %AO values obtained using the original algorithm in SB1 and duplicate measurements in SB2 analyzed with the optimized algorithm (Supplementary Figure 6), similar to our results using the transformation approach in QuPath (Supplementary Table 4 and Supplementary Figures 4, 5) and in Halo (Table 4 and Figure 5, 6). These findings suggest that digitally accounting for both haematoxylin and DAB provides for a comparable effect as our validated statistical transformation method (Figure 7).

## Discussion

Here we provide a statistically rigorous evaluation of pre-analytical variability in IHC staining intensity in FTLD (Figure 1), we develop an SOP for transformation of digital pathology measurements to account for this variability in both GM and WM (Figure 7), and we generalize our findings using a second, open-source, image analysis platform. First, Bland-Altman statistics suggests that variation in staining intensity is influential for measurements of digital pathology in both FTLD-Tau and FTLD-TDP in GM and WM (Figure 3), necessitating a method to transform values from different staining batches into equivalent values for more accurate analysis. Based on a highly correlated linear relationship between duplicate measurements of pathology from two different staining batches (Figure 4) in FTLD-Tau (GM: Rsq = 0.92, WM: Rsq = 0.92) and FTLD-TDP (GM: Rsq = 0.75, WM: Rsq = 0.78), we validate the use of a regression-based transformation method using a small set of tissue stained in duplicate to merge data obtained from different staining batches. First, we find that smaller datasets (*N* = 12–24) have adequate transformation prerequisites (i.e., Rsq), providing a consistently strong linear relationship (Table 2) to serve as training sets for our transformation protocol. Second, we find that training sets of *N* = 12 sample size in FTLD-Tau and *N* = 24 in FTLD-TDP result in optimal or near optimal transformation outcomes in complementary testing sets (Table 3). After applying our final transformation method, we observe a significant reduction in the difference between duplicate measurements from different staining batches in both FTLD-Tau (*p* < 0.001) and FTLD-TDP (*p* < 0.001) (Figure 5, 6). Finally, we perform a proof-of-concept power analysis, which shows that the application of our transformation method improves statistical power for analysis in both FTLD-Tau and FTLD-TDP, decreasing the required sample size by 20–40% (Table 5). Altogether, these results suggest that it is possible and advantageous to account for pre-analytical variability statistically, and this process can be performed using open-source platforms for greater rigor, reproducibility of digital measurements, and sharing of research methodologies. Therefore, these data have strong implications for digital pathology studies in neurodegenerative disease.

Digital measurements of pathology provide a novel and high-throughput means to obtain objective data of regional disease severity in the central nervous system of FTLD and related disorders. This approach allows for complex statistical modeling of quantitative pathology data for more fine-grained clinicopathological studies (Neltner et al., 2012; Hamilton et al., 2014; Walker et al., 2015; Coughlin et al., 2018; Ferman et al., 2018; Giannini et al., 2019). This is of importance as autopsy tissue remains the gold standard for diagnosis in neurodegenerative disease, and measurement of histopathological markers can inform biomarker discovery and validation. While clinicopathological studies have already been informative to improve the understanding of pathophysiological processes and guide clinical diagnostic criteria (Irwin et al., 2016a, 2017, 2018; Giannini et al., 2017), quantitative digital pathology has the potential to provide a more objective and detailed account of neuropathological burden, suitable for associations with biomarkers, imaging and other measures of disease (Irwin et al., 2018). Thus, a rigorous approach is needed to optimize digital pathology measurements for widespread use in the research community. We previously validated sampling methods and thresholding algorithms for FTLD (Irwin et al., 2016b), and successfully applied digital methods to relate *postmortem* FTLD histopathology to *antemortem* cerebrospinal fluid (CSF) (Irwin et al., 2017) and quantitative MRI data (Irwin et al., 2018; Giannini et al., 2019). We have also used this approach in Alzheimer’s disease (AD) and Lewy body disease (LBD) (Coughlin et al., 2018). Here, we validate a statistical methodology to account for an important pre-analytical factor in digital histopathology, similar to other approaches previously used for biofluid (Figurski et al., 2012) or neuroimaging (Cash et al., 2015) biomarkers, which helps us to account for staining batch effects in AO% measurements using a set of tissue stained in duplicate.

A unique aspect to digital pathology is the limited flexibility to stain large numbers of tissue samples in a single staining batch, which precludes the inclusion of large amounts of additional tissue as control sample for our transformation, as opposed to other biomarkers such as biofluid assays which often use multiple sets of >96 well plates. Therefore, our approach to determine a feasible number of tissue samples to use for this transformation method (i.e., ≤24, which is equivalent to a standard staining rack) is critical for implementation. We found relatively consistent goodness of fit (i.e., Rsq) in linear modeling derived from smaller trainings sets such as *N* = 12 or *N* = 24 in FTLD-Tau. FTLD-TDP showed relatively more heterogeneous transformation prerequisites (i.e., Rsq) in both GM and WM. We observed that lowering the number of samples in the training set increases the chance of a weaker linear relationship (Table 2). There may be several reasons for this observation. FTLD-Tau pathology has a wider magnitude and variance in overall %AO based on the morphology of tau inclusions, which in severe cases cover a large number of pixels (e.g., >70 %AO) (Irwin et al., 2017), compared to severe sections of FTLD-TDP, which cover a much smaller range of area (i.e., <5 %AO) (Irwin et al., 2018). The smaller variance in overall %AO in FTLD-TDP could potentially lead to an amplification of the effect of small changes in measurements between batches. Further, due to the small size of TDP-43 dystrophic neurites in GM and oligodendrocytic TDP-43 inclusions in WM (Neumann et al., 2009), biological variance in the amount of FTLD-TDP pathology in a given tissue sample may be influential even between adjacent sections. There may also be differences in antibody avidity or sensitivity to antigen retrieval, which could affect our outcomes focused on staining batch-related variability (Cummings et al., 2002). We used well-characterized antibodies (Goedert et al., 1995) with optimized staining parameters used in our lab and identical processing for both staining batches to reduce the influence of these factors.

It is well known that GM and WM have distinct densities and morphologies of disease in FTLD pathologies (Irwin et al., 2015). We therefore chose to analyze these two measures of disease separately in our validation analyses. In terms of transformation prerequisites and outcomes, we obtained relatively similar values between GM and WM within FTLD pathologies, indicating that these two measures of disease are affected by staining batch effects to a comparable extent. WM pathology in FTLD is important and understudied, especially using digital pathology methods. Indeed, in our most recent work we found greater overall burden of WM pathology in FTLD-Tau compared to FTLD-TDP (Irwin et al., 2018), which could help differentiate these pathologies during life (McMillan et al., 2013). While we now use a standardized sampling procedure (Irwin et al., 2017, 2018) of adjacent WM from cortical sections, future work will aim to explore sampling of specific deep WM tracks at the time of autopsy. Optimizing and validating pre-analytical methods for WM pathology analysis will be crucial to further the understanding of subcortical patterns of disease.

It is notable that the optimal size of training sets for an accurate transformation differed between FTLD-Tau and FTLD-TDP. These data suggest that transformation SOPs should be empirically determined for each disease (e.g., AD, LBD), and potentially for each antibody used in digital pathology studies. Here, we find optimal training-set sizes (i.e., ≥24) that are practical to use in prospective studies (i.e., requiring only half or one additional staining rack). It may be possible to further improve this transformation method using a larger number of training data by means of a tissue microarray slide with >100 pathology cores (Walker et al., 2017) in prospective staining runs, or tissue slides with standardized synthetic protein of interest for transformation (Sompuram et al., 2015). Our study provides important proof-of-concept findings for the use of linear regression to account for staining batch effects, thereby improving accuracy of digital histopathology, and new available tools may be used to facilitate and advance the implementation of this method.

Indeed, our method enabled us to correct for a large amount of variability due to staining batch effects (Figure 4, 5). We were additionally interested in the implications of our method to improve clinicopathological correlations. Using a power analysis to compare the effects of our transformation on merged data from distinct staining batches, we found a positive improvement in both FTLD-Tau and FTLD-TDP after transformation (Table 5). This is of great importance for FTLD, which is a relatively rare neurodegenerative disease (Knopman and Roberts, 2011), and may also be beneficial in other more common disorders to preserve valuable autopsy tissue and improve statistical power.

Our study proposes and validates a method to account for staining batch effects in digital histopathology, but has some limitations. Relying on a statistical estimation, our proposed method does not help to escape other individual-sample sources of variability in digital measurements, such as artifacts or damaged tissue. For this reason we performed rigorous inspection of all tissue sections used in this study and we carefully excluded those that were not of sufficient quality for usage and comparison across staining batches. Therefore, in the selection of tissue samples to use in prospective transformations, it is crucial to ensure that anomalous sections with observable defects are not included. While (semi-)adjacent tissue sections compared across staining batches seemed near-identical by visual inspection, biological variation in pathology distribution as well as ROI sampling between (semi-)adjacent tissue sections may partly confound the linear relationship between different staining batches measured in this study. However, the highly consistent linear relationship between the two staining batches (Figure 4) suggests that these effects may be minimal. Additionally, there may be other pre-analytical factors that influence digital pathology measurements that have not been accounted for by our designed methodology. While the intensity of haematoxylin counterstain may be variable and introduce further noise in the digital measurements, the Halo quantification accounted for the counterstain in color deconvolution algorithms (Supplementary Table 1). Moreover, in our supplementary analysis using QuPath, we empirically derived an optimized RGB color deconvolution algorithm for both haematoxylin and DAB in SB2 (Supplementary Table 5) with similar favorable results for test-retest agreement (Supplementary Figure 6) as in our transformation approach (Figure 7). We used a transmitted light microscopy scanning system with identical image acquisition features and resolution. Further work is needed to validate image acquisition across different scanners at different labs as another pre-analytic factor that may be optimized to increase the rigor and reproducibility of digital histopathology for multicenter studies in neurodegenerative disease (Rojo et al., 2003). Further, the use of other available methods of digital histopathology such as multispectral analysis may improve quantification (Van Der Loos, 2008). In an exploratory analysis, we suggested a potential application of our methodology to equate data with alternative fixation methods (i.e., fresh-fixed vs. frozen-fixed tissue), based on an observed consistent linear relationship between duplicate measurements from these two approaches (Supplementary Figure 2). These findings suggest that the use of our SOP may be extended to other identifiable sources of pre-analytical variability, granted that the divergence between digital measurements of pathology can be approximated to a linear relationship. Moreover, future targeted studies will be necessary to understand and address all potential sources of pre-analytical variability in digital histopathology systematically, and examine these variables carefully in large tissue samples with targeted study designs.

To conclude, we find that staining batch affects can significantly alter the accuracy of digital pathology measurements in neurodegenerative disease research. To account for this problem, we propose and validate a novel statistical approach using linear regression that enables to transform measurements from distinct staining batches into equivalent values, and to merge these data in a unique dataset without significant batch-related variability. Given the generalizability of our findings in an open-source digital pathology platform, we suggest that our method may provide a valid solution to researchers using different image analysis platforms. This approach will allow for more accurate and intercomparable measurements of digital pathology, and it will facilitate the creation of large-scale “libraries” of digital pathology data for future translational work.

## Ethics Statement

This study was carried out in accordance with the recommendations of the Penn Institutional Review Board (IRB) on human subjects research protections guidelines. The protocol was approved by the Penn IRB. All subjects gave written informed consent prior to participation, in accordance with the Declaration of Helsinki.

## Author Contributions

LG, SX, CZ, CM, and DI contributed to the conception and design of the study. LG, CP, and CZ organized the database. LG conducted the statistical analysis. LG and DI wrote the first draft of the manuscript. All authors contributed to the data acquisition, data analysis, manuscript revision, and also read and approved the submitted version.

## Conflict of Interest Statement

The authors declare that the research was conducted in the absence of any commercial or financial relationships that could be construed as a potential conflict of interest.
