# Supplementary material for: Empiric Methods to Account for Pre-analytical Variability in Digital Histopathology in Frontotemporal Lobar Degeneration
Source: Front Neurosci. 2019 Jul 3;13:682. doi: 10.3389/fnins.2019.00682 (PMC6616086; doi:10.3389/fnins.2019.00682)
Supplement: Supplementary file 1 [file Data_Sheet_1.docx]

Supplementary Material

# Supplementary Figures and Tables

## Supplementary Tables

## Supplementary Table 1. Halo detection algorithms for tau and TDP-43 immunohistochemical stain

| **Parameter** | **Tau %AO** | **TDP-43 %AO** |
| --- | --- | --- |
| Pathology Stain (RGB) | 0.614,1.176,0.975 | 0.296,0.433,0.493 |
| Counter Stain (RGB) | 0.369,0.433,0.159 | 0.313,0.313,0.097 |
| Stain Min. OD: | 0.146 | 0.182 |

Legend: Stain Min. OD = minimum OD value of DAB chromogen to differentiate pathology from background stain for % area occupied (%AO) calculation (% DAB positive pixels / total tissue pixels per ROI). Halo has additional parameters of minimum tissue OD to help distinguish signal of glass from tissue (Tau algorithm = 0.037, TDP algorithm = 0.028) and image magnification zoom for analysis (Tau algorithm = 1, TDP algorithm = 0.399).

**Supplementary Table 2. Summary statistics of duplicate measurements of pathology from two distinct staining batches**

|  | **SB1** | | **SB2** | | **SB1 vs. SB2** | | | **Linear regression** | | | |
| --- | --- | --- | --- | --- | --- | --- | --- | --- | --- | --- | --- |
|  | **ln %AO** | | **ln %AO** | | **(Bland-Altman)** | | |  |  |  |  |
|  | Mean | SD | Mean | SD | Mean diff | SD | Sig. | Rsq | Beta | Itc | Sig. |
| FTLD-Tau GM | 0.70 | 1.48 | -0.43 | 1.6 | 1.13 | 0.44 | < 2.2e-16 | 0.92 | 1.08 | 0.89 | < 2.2e-16 |
| N = 39 |  |  |  |  |  |  |  |  |  |  |  |
| FTLD-Tau WM | -0.37 | 1.86 | -1.65 | 1.98 | 1.28 | 0.56 | < 2.2e-16 | 0.92 | 0.9 | 1.11 | < 2.2e-16 |
| N = 39 |  |  |  |  |  |  |  |  |  |  |  |
| FTLD-TDP GM | -1.83 | 1.34 | -2.78 | 1.17 | 0.95 | 0.66 | 1.93e-14 | 0.75 | 1.00 | 0.96 | < 2.2e-16 |
| N = 53 |  |  |  |  |  |  |  |  |  |  |  |
| FTLD-TDP WM | -3.25 | 1.5 | -4.15 | 1.64 | 0.90 | 0.77 | 1.86e-11 | 0.78 | 0.81 | 0.09 | < 2.2e-16 |
| N = 53 |  |  |  |  |  |  |  |  |  |  |  |

Legend: diff = difference; FTLD-Tau = frontotemporal lobar degeneration with inclusions of the tau protein; FTLD-TDP = frontotemporal lobar degeneration with inclusions of the transactive response DNA-binding protein 43 kDa; GM = grey matter; Itc = intercept; ln %AO = natural logarithmic transformation of percent of area occupied by pathology (%AO); Rsq = R squared; SD = standard deviation; SB1 = staining batch 1 (original); SB2 = staining batch 2 (new); Sig. = significance; WM = white matter. Table shows summary statistics for duplicate pathology data from SB1 and SB2 in FTLD-Tau and FTLD-TDP, separately for GM and WM. We further display Bland-Altman statistics (i.e. one-sided t-test, null hypothesis: mean difference = 0) demonstrating highly significant difference in digital measurements between SB1 and SB2, and univariate linear regression models showing a significant linear relationship between SB1 and SB2 data in the entire FTLD-Tau and FTLD-TDP datasets.

**Supplementary Table 3. Summary statistics of duplicate measurements of pathology from two distinct staining batches in QuPath**

|  | **SB1** | | **SB2** | | **SB1 vs. SB2** | | | **Linear regression** | | | |
| --- | --- | --- | --- | --- | --- | --- | --- | --- | --- | --- | --- |
|  | **ln %AO** | | **ln %AO** | | **(Bland-Altman)** | | |  |  |  |  |
|  | Mean | SD | Mean | SD | Mean diff | SD | Sig. | Rsq | Beta | Itc | Sig. |
| FTLD-Tau GM | 1.01 | 1.87 | -0.05 | 1.88 | 1.06 | 0.52 | 5.68E-15 | 0.92 | 0.95 | 1.06 | < 2.2e-16 |
| N = 38 |  |  |  |  |  |  |  |  |  |  |  |
| FTLD-Tau WM | -0.29 | 2.15 | -1.78 | 2.29 | 1.48 | 1.00 | 5.55E-11 | 0.80 | 0.84 | 1.20 | < 2.2e-16 |
| N = 38 |  |  |  |  |  |  |  |  |  |  |  |
| FTLD-TDP GM | -1.56 | 1.28 | -2.49 | 1.36 | 0.93 | 0.81 | 6.78E-11 | 0.66 | 0.77 | 0.36 | 3.17E-13 |
| N = 51 |  |  |  |  |  |  |  |  |  |  |  |
| FTLD-TDP WM | -3.03 | 1.54 | -3.82 | 1.36 | 0.79 | 0.87 | 2.78E-08 | 0.73 | 0.80 | 0.02 | 7.54E-16 |
| N = 52 |  |  |  |  |  |  |  |  |  |  |  |

Legend: diff = difference; FTLD-Tau = frontotemporal lobar degeneration with inclusions of the tau protein; FTLD-TDP = frontotemporal lobar degeneration with inclusions of the transactive response DNA-binding protein 43 kDa; GM = grey matter; Itc = intercept; ln %AO = natural logarithmic transformation of percent of area occupied by pathology (%AO); Rsq = R squared; SD = standard deviation; SB1 = staining batch 1 (original); SB2 = staining batch 2 (new); Sig. = significance; WM = white matter. Table shows summary statistics for duplicate pathology measurements from SB1 and SB2 in FTLD-Tau and FTLD-TDP, tested using an open-source digital pathology platform (i.e. QuPath). We display Bland-Altman statistics (i.e. one-sided t-test, null hypothesis: mean difference = 0) demonstrating highly significant difference in digital measurements between SB1 and SB2, and univariate linear regression models showing a significant linear relationship between SB1 and SB2 data in the entire FTLD-Tau and FTLD-TDP datasets. These results in QuPath are comparable to our previous findings using the Halo software, showing generalizability of staining batch effects across digital pathology platforms.

**Supplementary Table 4. Application of transformation method in a single random train-test split to determine transformation outcomes in QuPath**

|  | **Train (N)** | **Rsq** | **Beta** | **Itc** | **Test (N)** | **ICC** | **Mean diff before** | **BA before sig.** | **Mean diff after** | **BA after sig.** | **Delta abs- diff** | **Delta abs-diff sig.** |
| --- | --- | --- | --- | --- | --- | --- | --- | --- | --- | --- | --- | --- |
| **1x train-test split FTLD-Tau** | | | | |  | | | | | | | |
| **GM** | 12 | 0.90 | 0.92 | 1.02 | 26 | 0.96 | -1.09 | 5.16E-12 | -0.05 | 0.58 | -0.71 | 2.61E-08 |
| **WM** | 12 | 0.81 | 0.69 | 1.07 | 26 | 0.82 | -1.49 | 1.76E-09 | 0.21 | 0.31 | -0.68 | 3.99E-03 |
| **1x train-test split FTLD-TDP** | | | | |  | | | | | | | |
| **GM** | 24 | 0.59 | 0.62 | 0.07 | 27 | 0.74 | -0.92 | 1.09E-05 | 0.15 | 0.40 | -0.45 | 0.02 |
| **WM** | 24 | 0.66 | 0.79 | -0.17 | 28 | 0.86 | -0.98 | 2.15E-06 | -0.27 | 0.09 | -0.35 | 0.01 |

Legend: BA = Bland-Altman statistics; Delta abs-diff = change in absolute difference; diff = difference; FTLD-Tau = frontotemporal lobar degeneration with inclusions of the tau protein; FTLD-TDP = frontotemporal lobar degeneration with inclusions of the transactive response DNA-binding protein 43 kDa; ICC = intraclass correlation coefficient; Itc = intercept; GM = grey matter; N = number of tissue samples; Rsq = R squared; sig. = significance; WM = white matter. Here, we display the results of the application of our transformation method in a single random train-test split of datasets analyzed in an open-source digital platform (i.e. QuPath). On the left side, table shows transformation prerequisites (i.e. Rsq) and equivalence factors (i.e. beta, intercept) in training sets (GM/WM in FTLD-Tau and FTLD-TDP). On the right side, we report corresponding transformation outcomes in the complementary testing sets, including ICC and measures of test-retest agreement (i.e. Bland-Altman statistics, Delta abs-diff). These results in QuPath are comparable to our previous findings using the Halo software, showing generalizability of our transformation method across digital pathology platforms.

## Supplementary Table 5. QuPath optimized detection algorithms for DAB chromogen and haematoxylin counterstain in SB2

| **Parameter** | **Tau %AO** | **TDP-43 %AO** |
| --- | --- | --- |
| Pathology Stain (RGB) | 0.453, 0.629, 0.575 | 0.362, 0.611, 0.6235 |
| Counter Stain (RGB) | 0.658, 0.725, 0.197 | 0.656, 0.723, 0.211 |
| Stain Min. OD: | 0.117 | 0.169 |

Legend: OD = Optical density. SB2 = Staining batch 2. RGB values in QuPath are on a range of 0-1 (0 = white, 1 = black) values. QuPath has additional parameters of downsampling (comparable to image zoom for analysis) and “Gaussian Sigma” smoothing function: FTLD-Tau algorithm downsample = 2, Gaussian Sigma = 1 μm; FTLD-TDP algorithm downsample = 2, Gaussian Sigma = 0.2 μm.

## Supplementary Figures


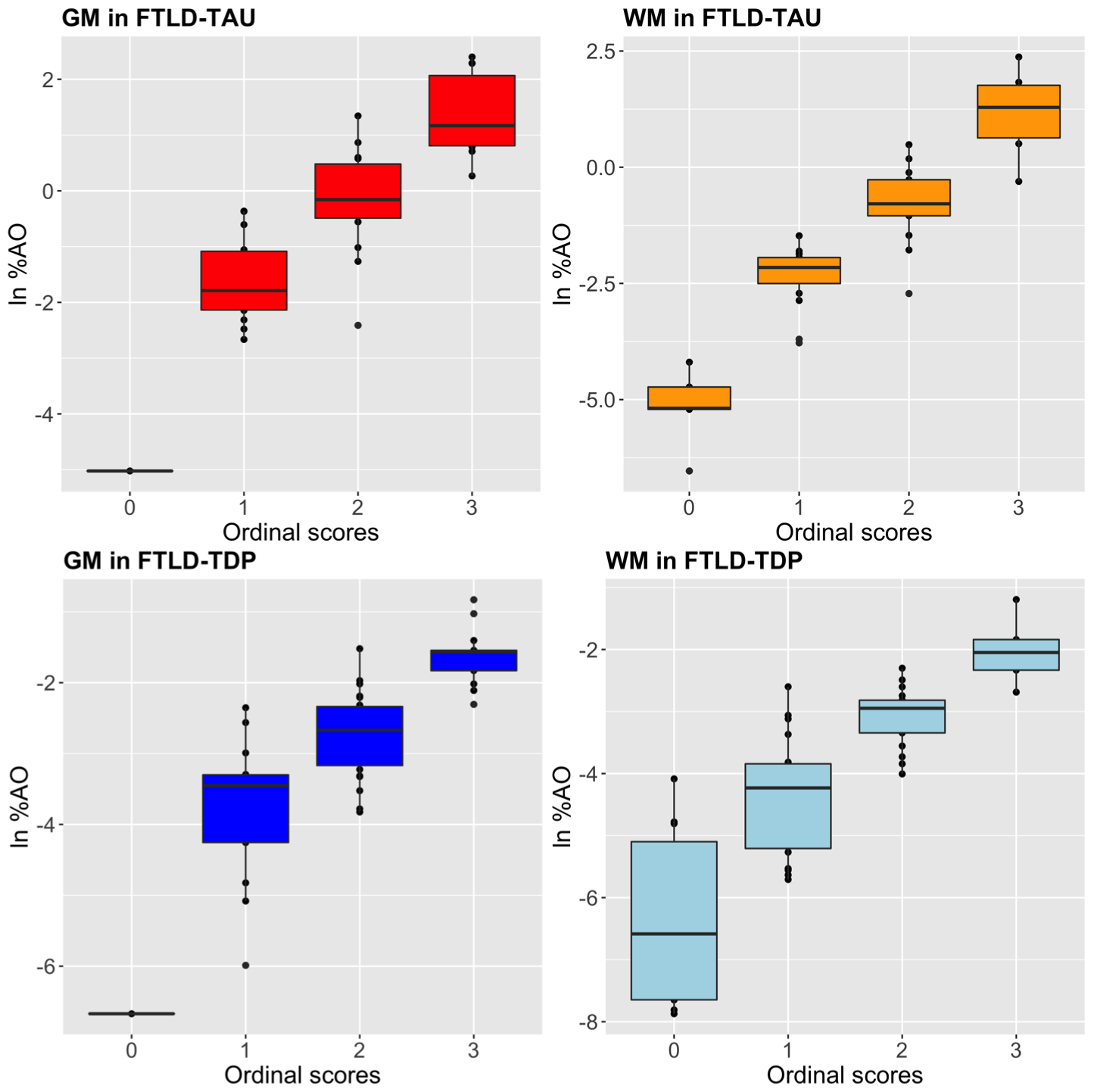
**Supplementary Figure 1. Validation of digital pathology measurements by comparison to gold-standard ordinal ratings**Plots portray the relationship between conventional ordinal ratings of pathology (i.e. 0-3 scores for each proteinopathy) and digitally acquired ln %AO scores from the original staining batch (SB-1) in FTLD-Tau (above) and FTLD-TDP (below). ANOVA analysis finds that ordinal ratings are significantly associated with digital pathology %AO scores in FTLD-Tau GM (F = 34.4, df = 3,35, p < 0.001) and WM (F = 64.7, df = 3,35, p < 0.001), and in FTLD-TDP GM (F = 31.8, df = 3,47, p < 0.001) and WM (F = 38.1, df = 3,47, p < 0.001). Legend: FTLD-Tau = frontotemporal lobar degeneration with inclusions of the tau protein; FTLD-TDP = frontotemporal lobar degeneration with inclusions of the transactive response DNA-binding protein 43 kDa; GM = grey matter; ln AO% = natural logarithmic transformation of percent of area occupied by pathology (%AO); WM = white matter.

**
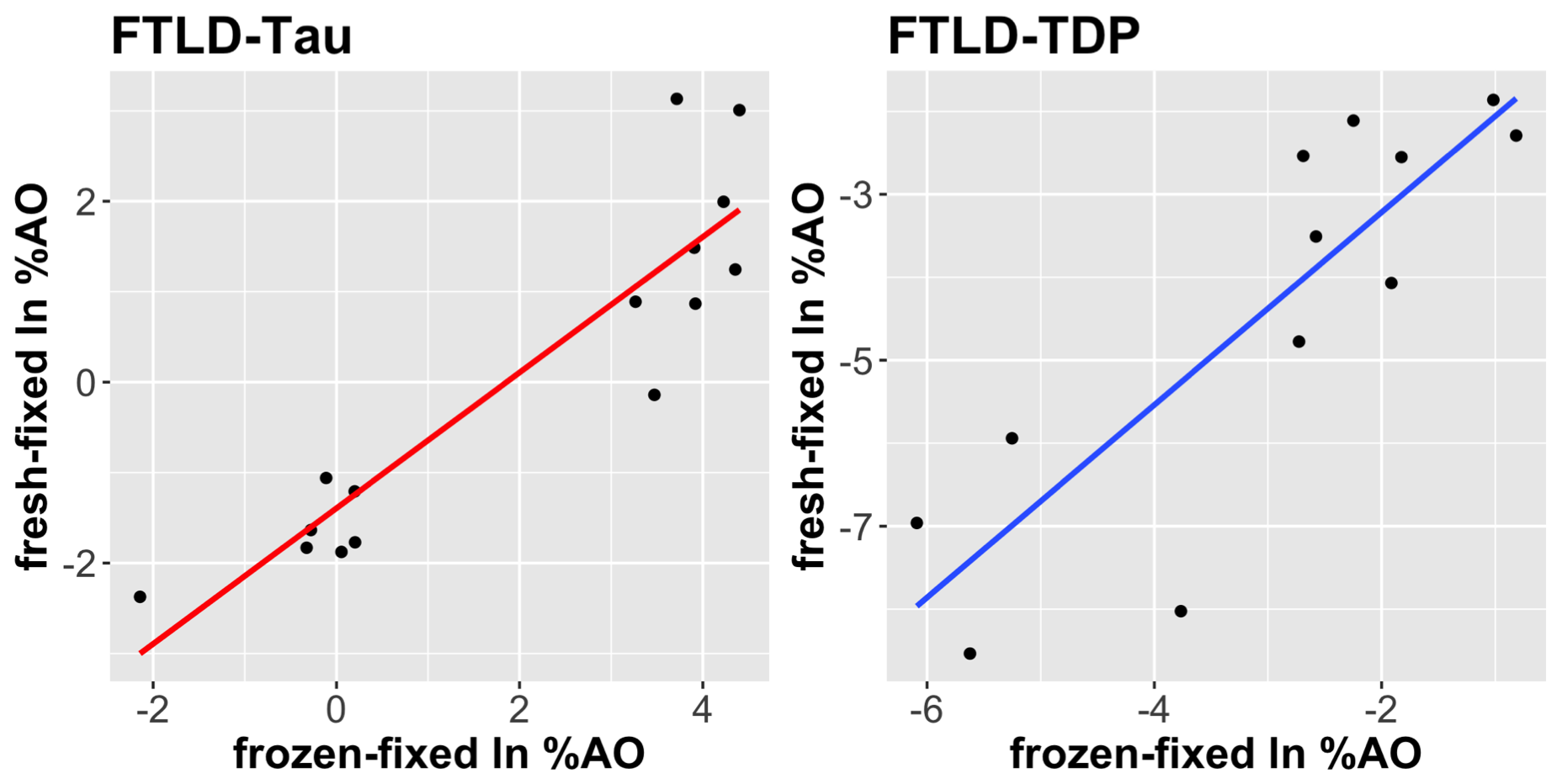
**

**Supplementary Figure 2. Potential application of our transformation method to other sources of pre-analytical variability (fresh-fixed vs. frozen-fixed tissue)**We attempted to apply our transformation method to see whether it could help account for another additional source of pre-analytical variability, i.e. fixation of tissue directly after autopsy (i.e. fresh-fixed) as opposed to fixation of tissue after being stored frozen at -80 degrees since autopsy (i.e. frozen-fixed). For this purpose, we obtained tissue from frozen coronal slabs in an identical manner to fresh sampling done at autopsy along with a small set of duplicate tissue samples from MFC for equivalence analyses (N = 16 in FTLD-Tau, N = 12 in FTLD-TDP). We tested whether frozen-fixed and fresh-fixed data were linearly related using a linear regression relating these duplicate measurements, using fresh-fixed data as dependent variable and frozen-fixed data as independent variable. Linear regression found a significant linear association between frozen-fixed and fresh-fixed duplicate measurements of pathology in both FTLD-Tau (Rsq = 0.77, p < 0.001) and FTLD-TDP (Rsq = 0.70, p < 0.001). The resulting linear equivalence factors were: beta = 0.79 and intercept = -1.62 in FTLD-Tau, beta = 1.16 and intercept = -0.89 in FTLD-TDP. Legend: FTLD-Tau = frontotemporal lobar degeneration with inclusions of the tau protein; FTLD-TDP = frontotemporal lobar degeneration with inclusions of the transactive response DNA-binding protein 43 kDa; ln %AO = natural logarithmic transformation of percent of area occupied by pathology (%AO).


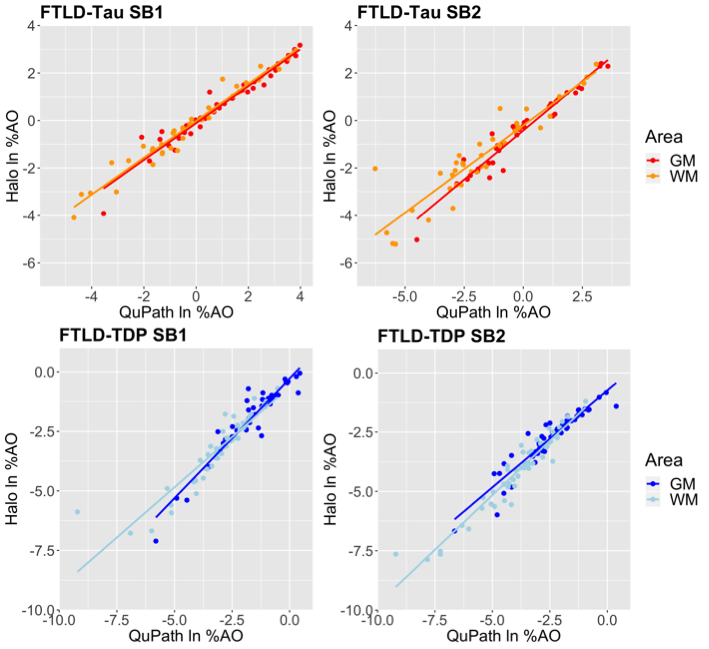


**Supplementary Figure 3. Linear relationship between %AO measurements across digital platforms in Halo and QuPath**Plots portray the strong association between %AO measurements in identical images analyzed using two different digital pathology platforms, i.e. Halo and QuPath. In FTLD-Tau, there is a significant linear relationship between Halo and QuPath measurements in both SB1 (GM Rsq = 0.94, p < 0.001; WM Rsq = 0.96, p < 0.001) and SB2 (GM Rsq = 0.94, p < 0.001; WM Rsq = 0.84, p < 0.001). Similarly, in FTLD-TDP there is a significant linear relationship between Halo and QuPath measurements in both SB1 (GM Rsq = 0.90, p < 0.001; WM Rsq = 0.84, p < 0.001) and SB2 (GM Rsq = 0.88, p < 0.001; WM Rsq = 0.92, p < 0.001). Legend: FTLD-Tau = frontotemporal lobar degeneration with inclusions of the tau protein; FTLD-TDP = frontotemporal lobar degeneration with inclusions of the transactive response DNA-binding protein 43 kDa; GM = grey matter; ln AO% = natural logarithmic transformation of percent of area occupied by pathology (%AO); WM = white matter.


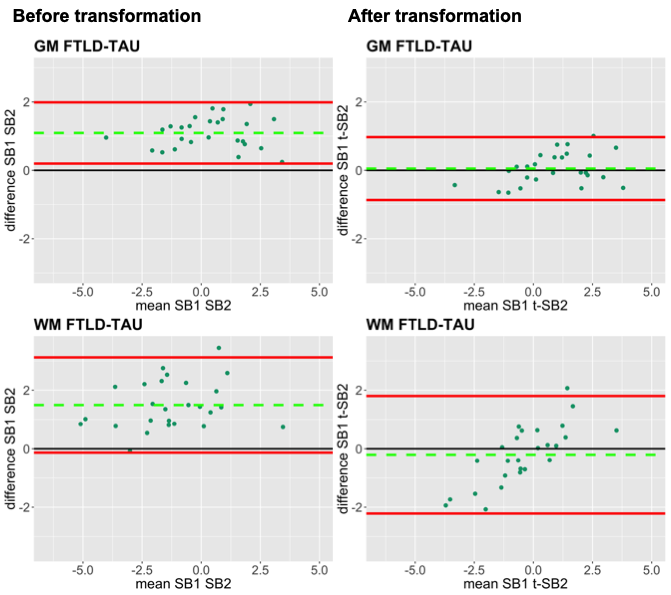


**Supplementary Figure 4. Bland-Altman plots of test-retest agreement between duplicate measurements of FTLD-Tau pathology before vs. after transformation in QuPath**Plots portray test-retest agreement between duplicate measurements of digital pathology (i.e. ln %AO) in FTLD-Tau before and after transforming the data obtained from an open source digital platform, i.e. QuPath. Here we illustrate the reduction in batch-related difference in digital measurements resulting from the application of our transformation method in a single train-test split in FTLD-Tau (see Supplementary Table 4). The green dashed line indicates the mean difference between SB1 and SB2 measurements, while the red solid lines mark the 95% limits of agreement between the two measurements. We find that mean difference between SB1 and SB2/t-SB2 is significantly different from zero before transformation (p < 0.05, one-sample t-test), whereas it does not differ from zero after transformation (p > 0.05) in both GM and WM. These results in QuPath are in agreement with our previous findings in the Halo software using the same detection algorithms for both batches. Legend: FTLD-Tau = frontotemporal lobar degeneration with inclusions of the tau protein; GM = grey matter; SB1 = staining batch 1 (original); SB2 = staining batch 2 (new); t-SB2 = transformed staining batch 2 (new); WM = white matter.

**
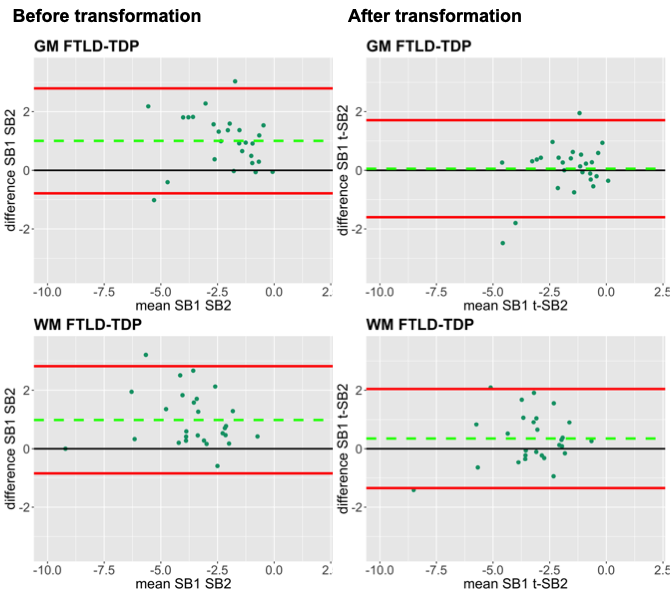
**

**Supplementary Figure 5. Bland-Altman plots of test-retest agreement between duplicate measurements of FTLD-TDP pathology before vs. after transformation in QuPath**Plots portray test-retest agreement between duplicate measurements of digital pathology (i.e. ln %AO) in FTLD-TDP before and after transforming the data obtained from an open source digital platform, i.e. QuPath. Here we illustrate the reduction in batch-related difference in digital measurements resulting from the application of our transformation method in a single train-test split in FTLD-TDP (see Supplementary Table 4). The green dashed line indicates the mean difference between SB1 and SB2 measurements, while the red solid lines mark the 95% limits of agreement between the two measurements. We find that mean difference between SB1 and SB2/t-SB2 is significantly different from zero before transformation (p < 0.05, one-sample t-test), whereas it is not significantly different from zero after transformation (p > 0.05) in both GM and WM. These results in QuPath are in agreement with our previous findings in the Halo software using the same detection algorithms for both batches. Legend: FTLD-TDP = frontotemporal lobar degeneration with inclusions of the transactive response DNA-binding protein 43; GM = grey matter; SB1 = staining batch 1 (original); SB2 = staining batch 2 (new); t-SB2 = transformed staining batch 2 (new); WM = white matter.

**
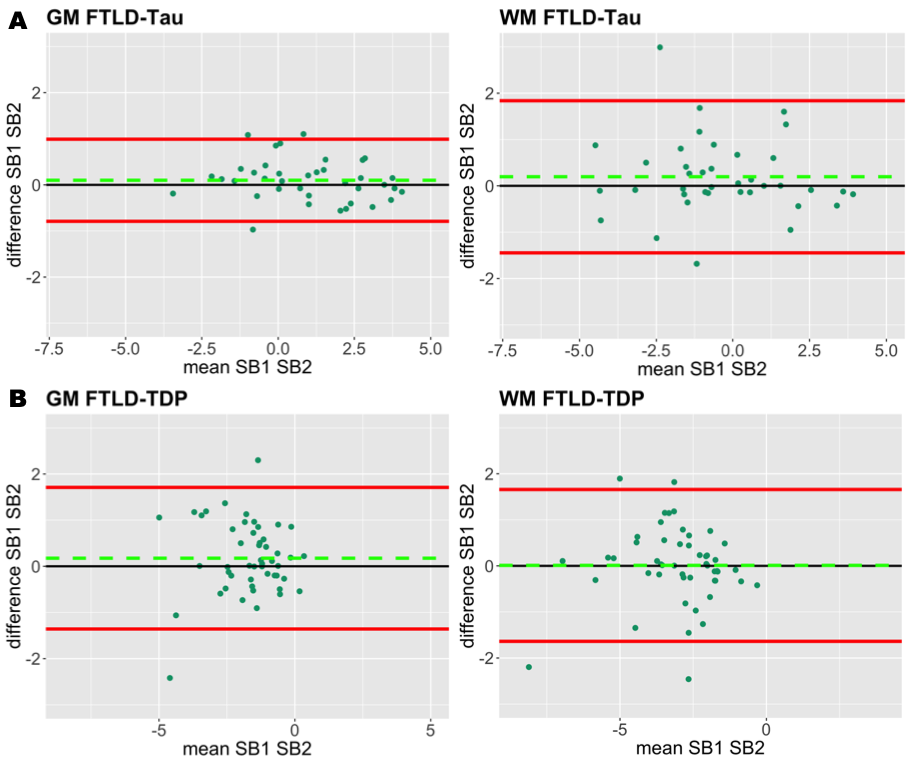
**

**Supplementary Figure 6. Bland-Altman plots of test-retest agreement between duplicate measurements of pathology comparing SB1 to SB2-optimized algorithms derived in QuPath**Bland-Altman plots show test-retest agreement between SB1 and SB2 measurements of digital pathology (i.e. ln %AO). The green dashed line indicates the mean difference between SB1 and SB2 measurements, while the red solid lines mark the 95% limits of agreement between the two measurements. SB1 has been analyzed in QuPath using standard algorithms, whereas SB2 has been analyzed in QuPath using optimized algorithms to account for potential variation in both counterstain and DAB chromogen. These algorithms were derived using the “Estimate stain vectors” function in QuPath (see detailed explanation in the Methods). We compared SB1 to optimized SB2 measurements and found that the mean difference between staining batches was not significantly different from zero in both FTLD-Tau (GM = 0.09, p = 0.183; WM = 0.01, p = 0.158) and FTLD-TDP (GM = 0.17, p = 0.124; WM = 0.01; p = 0.931), as opposed to the large mean difference between staining batches using a single algorithm for both batches in Supplementary Figures 4-5 (“Before transformation” panels) . Legend: FTLD-Tau = frontotemporal lobar degeneration with inclusions of the tau protein; FTLD-TDP = frontotemporal lobar degeneration with inclusions of the transactive response DNA-binding protein 43 kDa; GM = grey matter; SB1 = staining batch 1 (original); SB2 = staining batch 2 (new); WM = white matter.
